# Expanding community case management of malaria to all ages can improve universal access to malaria diagnosis and treatment: results from a cluster randomized trial in Madagascar

**DOI:** 10.1186/s12916-024-03441-9

**Published:** 2024-06-10

**Authors:** Andres Garchitorena, Aina Harimanana, Judickaelle Irinantenaina, Hobisoa Léa Razanadranaivo, Tsinjo Fehizoro Rasoanaivo, Dean Sayre, Julie R. Gutman, Reziky Tiandraza Mangahasimbola, Masiarivony Ravaoarimanga, Oméga Raobela, Lala Yvette Razafimaharo, Nicolas Ralemary, Mahefa Andrianasolomanana, Julie Pontarollo, Aline Mukerabirori, Walter Ochieng, Catherine M. Dentinger, Laurent Kapesa, Laura C. Steinhardt

**Affiliations:** 1 UMR MIVEGEC, Université de Montpellier, IRD, CNRS, Montpellier, France; 2 Unité d’épidémiologie et de recherche clinique, Institut Pasteur de Madagascar, Antananarivo, Madagascar; 3 U.S. President’s Malaria Initiative, Malaria Branch, Centers for Disease Control and Prevention, Atlanta, GA USA; 4 Malaria Branch, Division of Parasitic Diseases and Malaria, Centers for Disease Control and Prevention, Atlanta, GA USA; 5 Programme National de Lutte contre le Paludisme, Ministère de la Santé Publique de Madagascar, Antananarivo, Madagascar; 6 Direction Régionale de la Santé, Ministère de la Santé Publique, Farafangana, Madagascar; 7 Bureau de Santé du District, Ministère de la Santé Publique, Farafangana, Madagascar; 8 ONG Inter Aide, Antananarivo, Madagascar; 9 Management Sciences for Health, Antananarivo, Madagascar; 10 Global Health Center, Centers for Disease Control and Prevention, Atlanta, GA USA; 11 U.S. President’s Malaria Initiative, US Centers for Disease Control and Prevention, Antananarivo, Madagascar; 12 U.S. President’s Malaria Initiative, USAID, Antananarivo, Madagascar

**Keywords:** Community health, Geographic access to care, Last mile interventions, Health systems strengthening, Supply chain

## Abstract

**Background:**

Global progress on malaria control has stalled recently, partly due to challenges in universal access to malaria diagnosis and treatment. Community health workers (CHWs) can play a key role in improving access to malaria care for children under 5 years (CU5), but national policies rarely permit them to treat older individuals. We conducted a two-arm cluster randomized trial in rural Madagascar to assess the impact of expanding malaria community case management (mCCM) to all ages on health care access and use.

**Methods:**

Thirty health centers and their associated CHWs in Farafangana District were randomized 1:1 to mCCM for all ages (intervention) or mCCM for CU5 only (control). Both arms were supported with CHW trainings on malaria case management, community sensitization on free malaria care, monthly supervision of CHWs, and reinforcement of the malaria supply chain. Cross-sectional household surveys in approximately 1600 households were conducted at baseline (Nov–Dec 2019) and endline (Nov–Dec 2021). Monthly data were collected from health center and CHW registers for 36 months (2019–2021). Intervention impact was assessed via difference-in-differences analyses for survey data and interrupted time-series analyses for health system data.

**Results:**

Rates of care-seeking for fever and malaria diagnosis nearly tripled in both arms (from less than 25% to over 60%), driven mostly by increases in CHW care. Age-expanded mCCM yielded additional improvements for individuals over 5 years in the intervention arm (rate ratio for RDTs done in 6–13-year-olds, RR_RDT6–13 years_ = 1.65; 95% CIs 1.45–1.87), but increases were significant only in health system data analyses. Age-expanded mCCM was associated with larger increases for populations living further from health centers (RR_RDT6–13 years_ = 1.21 per km; 95% CIs 1.19–1.23).

**Conclusions:**

Expanding mCCM to all ages can improve universal access to malaria diagnosis and treatment. In addition, strengthening supply chain systems can achieve significant improvements even in the absence of age-expanded mCCM.

**Trial registration:**

The trial was registered at the Pan-African Clinical Trials Registry (#PACTR202001907367187).

**Supplementary Information:**

The online version contains supplementary material available at 10.1186/s12916-024-03441-9.

## Background

Despite ambitious targets for malaria control and elimination, annual global malaria cases are estimated to have increased by 17 million from 2015 to 2021 [1]. Ensuring universal access to malaria diagnosis and treatment is a key pillar of the global malaria strategic plan for 2016–2030 [2], but access remains limited in sub-Saharan Africa (SSA), a region that bears 95% of the global malaria burden [1]. Community health workers (CHWs) can play a critical role in expanding access to care, especially in rural and more remote areas [3]. However, CHWs typically only diagnose and treat children under five years of age (CU5) as part of integrated community case management (iCCM), a strategy initially recommended by UNICEF and WHO to reduce mortality from the most common childhood illnesses: malaria, pneumonia, and diarrhea [4]. Previous studies have shown that CHWs can effectively manage patients for these diseases [5–7] and that they can improve access to quality care for CU5 [8]. With evidence of CHWs’ ability to extend the reach of the health system, multiple efforts are trying to expand the scope of their work [9–11].

Beyond child-focused interventions, CHWs can play an important role in diagnosing and treating malaria cases of all ages. This is already the case in many countries pursuing malaria elimination nationally or sub-nationally [12]. In moderate to high transmission settings, engaging CHWs in efforts to visit homes at regular intervals, identify febrile household members, and test and treat them according to a standard protocol, a strategy known as proactive community case management (pro-CCM), has shown some success in increasing malaria cases detected [13, 14] and improving malaria outcomes [15, 16]. However, pro-CCM approaches can be time- and resource-consuming for CHWs, who are often volunteers in many SSA countries due in part to a lack of funding for community health programs. As a less resource-intensive alternative, several countries have expressed interest in expanding malaria community case management (mCCM) to older children and adults, but few countries have formally adopted this policy to date. Rigorous evaluation of the age expansion of mCCM has been limited, although an initial analysis in Rwanda suggested that the incidence of severe malaria in areas where CHWs provided mCCM to all individuals was lower than in those where mCCM was restricted to CU5 during a malaria upsurge, presumably due to increased access to prompt and effective malaria case management [17].

Expanding mCCM to individuals over five years of age was included in the 2018–2022 National Malaria Strategic Plan in Madagascar [18]. The country has seen a surge in malaria cases in recent years, with both malaria incidence and mortality increasing by over 75% between 2015 and 2021 [1]. Malaria transmission on this island-nation off the southeastern coast of Africa is heterogeneous, with an average national prevalence in children under 5 years of 7.5% in 2021 that ranged from very low in the highlands (< 1% prevalence) to high transmission in many coastal areas (> 20% prevalence) [19, 20]. Madagascar has a network of approximately 36,000 CHWs who provide iCCM services to CU5, as well as health prevention and promotion services to communities, among other activities [21]. The population coverage target set by the Madagascar Ministry of Public Health (MoPH) is one CHW per 1000 individuals, and there are generally two CHWs in every *fokontany*, the smallest administrative unit in Madagascar comprising one or several villages. CHWs are not formally paid, although some receive incentives for attending trainings or monthly meetings at health centers, or for participating in campaigns outside their regular duties (e.g., bed net distribution, mass drug administration, vaccination). Although malaria services are officially free, CHWs are authorized to earn money from the sale of other health commodities and treatments [22]. Prior to a policy shift that would allow CHWs to diagnose and treat febrile people of all ages for malaria across the country, assessing the effectiveness of expanding mCCM to older ages in one pilot district was deemed necessary by the National Malaria Control Program (NMCP) and its partners. This cluster-randomized study was undertaken in a rural district in southeastern Madagascar to assess the impact of expanding mCCM to all ages in improving access to and use of malaria case management services.

## Methods

### Study area

Farafangana is a coastal district in the Atsimo Atsinanana Region in south-eastern Madagascar with a population of approximately 400,000 individuals, 90% of whom live in rural areas [23]. Farafangana has 38 public health facilities and over 600 CHWs, who receive supplies and supervision during monthly visits to their supervising health facility. Malaria transmission in the district varies seasonally, with increased transmission during the rainy season from October to April. Passive surveillance data from 2015 to 2017 indicated an average annual incidence of nearly 100 cases per 1000 population [24]. Farafangana benefits from long-term support from the non-governmental organization (NGO) Inter Aide, which has strengthened community health activities for over ten years through monthly supervision efforts, data quality reviews, training of CHWs on iCCM, and community sensitization on a range of health issues, including malaria. Despite regular mass distribution campaigns of long-lasting insecticide-treated bed nets and indoor residual spraying campaigns (before the study, the latest was in 2018 with Actellic® 300CS), Farafangana continued to have high levels of malaria transmission when the study was implemented in 2019.

### Study design

**Fig. 1:**
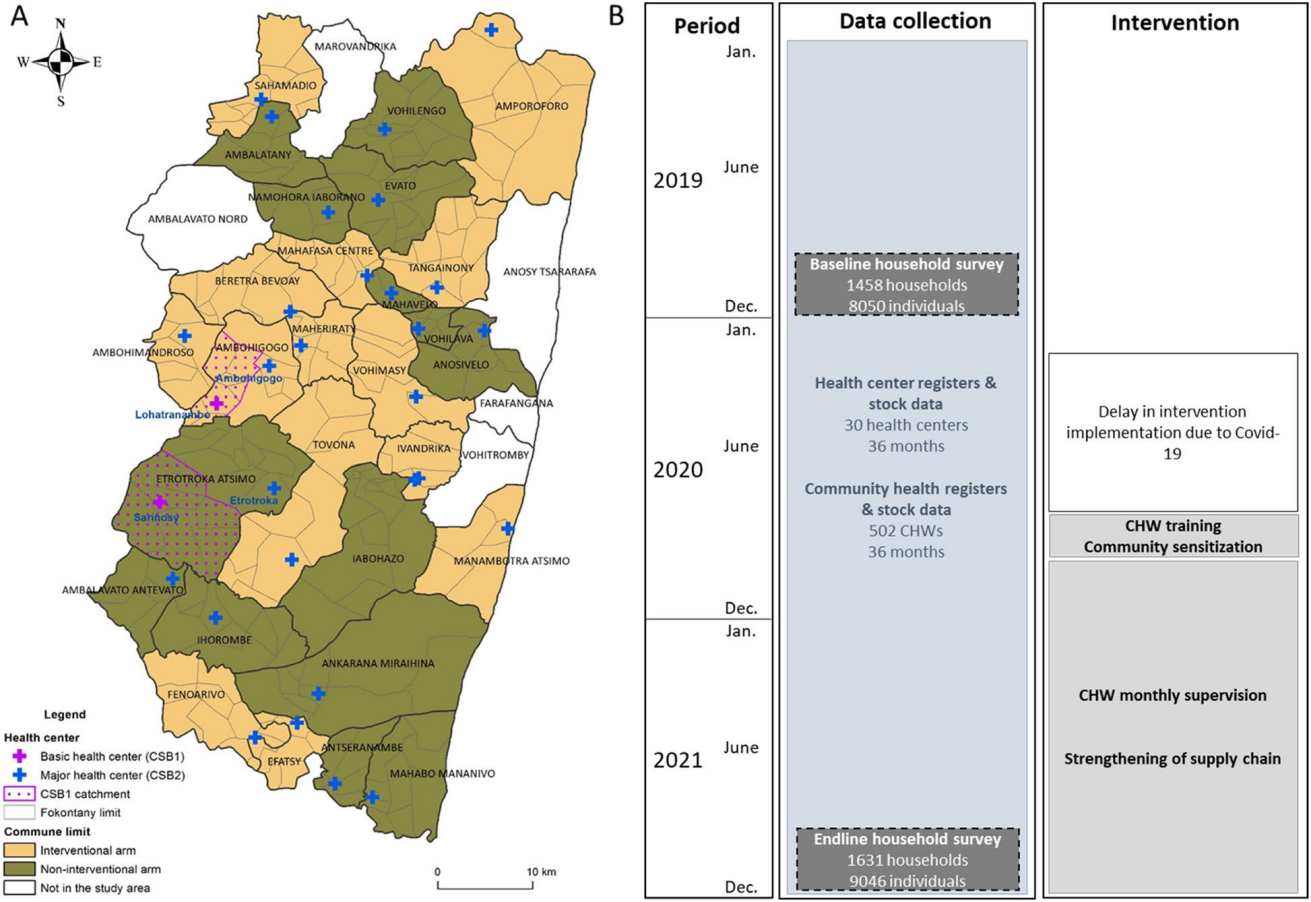
Study design of mCCM cluster randomized trial in Farafangana District. **A** Map of Farafangana district and the health center catchments randomized to the intervention (yellow) and control arms (green). **B** Summary diagram of data collected at baseline, follow-up, and endline, and main intervention activities implemented. Note: in addition to household surveys, qualitative information was gathered at endline via individual interviews and focus groups (results presented in a separate manuscript)

The study was a two-arm cluster-randomized intervention trial, randomizing 15 health facilities with their CHW catchment areas to age-expanded mCCM (intervention arm) and 15 health facilities with their CHW catchment areas to standard mCCM for CU5 only (control arm) (Fig. 1). Non-rural health facilities were excluded from the study. In both arms, CU5 had access to iCCM through CHWs in their *fokontany*, and individuals of all ages had access to malaria case management at the nearest health facility, corresponding to the current national policy. The main objective was to evaluate the impact that the expansion of mCCM to all age groups (referred here as age-expanded mCCM) had on rates of care-seeking for fever, and malaria diagnosis and treatment in the study area. Health facilities were assigned by the study team to intervention and control groups using restricted randomization to ensure malaria prevalence in children and rates of care-seeking were balanced between arms. For this, a random subset consisting of 500,000 of the 1.55 × 10^8^ possible combinations was generated. For each member of this subset, aggregate group malaria prevalence estimates in children and household-level care-seeking estimates generated during the baseline survey were calculated. For the purposes of this calculation, household-level care-seeking was dichotomized into those with a member seeking care from a health facility or community health worker within the past month and those without a member seeking care from these sources within the past month. The final study assignment was randomly selected from the schemes within the subset (4.2% of the 500,000 combinations) having matching malaria prevalence in intervention and control groups (+ / − 0.01) and matching household-level care-seeking estimates (+ / − 0.02).

### Outcome measures, hypotheses, and sample size

Our primary outcome was the proportion of individuals 2 months of age or older reporting a fever in the previous 2 weeks who were tested with a malaria RDT by a CHW or at a health facility by a health worker. Secondary outcome measures were:

- Proportion of individuals 2 months of age or older reporting a fever in the previous 2 weeks who (i) sought care for that illness and (ii) if tested positive for malaria, received treatment with an appropriate antimalarial.
- Proportion of children < 5 years, children 5–14 years, and those aged 15 + years with febrile illness in the previous 2 weeks who (i) sought care for that illness, (ii) were tested for malaria with a malaria RDT, and (iii) were treated with an appropriate antimalarial if tested positive for malaria.
- Community-level parasite prevalence in children under 15 years (including subgroup analyses for < 5 years and 5–14 years) of age as measured by malaria RDT.
- Proportion of children < 5 years with suspected pneumonia and diarrhea in the previous 2 weeks who sought care and who received appropriate treatment.

These outcomes were evaluated through difference-in-difference analyses of cross-sectional household survey data (primary analysis), and through interrupted time-series analyses with control groups of equivalent health system data from all health facilities and CHWs in the study area (except for malaria prevalence). Both sets of analyses were pre-specified in the protocols approved prior to the beginning of the trial. Details on data collection and analysis for each data source are available in the corresponding sections below. A detailed description of each outcome measure is available in the Additional file 1: Table S1.

We hypothesized that age-expanded mCCM would lead to an increase in care-seeking by patients with fever, in the proportion of subjects with fever who received a malaria RDT, and in the proportion of cases of malaria confirmed by RDT who received adequate antimalarial treatment. More specifically, for our sample size calculation for the primary outcome (proportion of people with fever in the last 2 weeks who are tested for malaria by a CHW or at a health facility by a health worker) for the cross-sectional surveys, we assumed that on average, 56% of households would have a respondent reporting a fever in the past 2 weeks and that 18% of febrile people are tested for malaria at baseline. Assuming an intraclass correlation coefficient of 0.1 for the primary outcome and an increase in the proportion of people with fever who are tested for malaria from 18 to 22.5% in the control arm, and from 18 to 39.5% in the group with age-expanded mCCM (17 percentage point difference at endline), 80% power, and assuming a 15% non-response rate, we estimated that we needed 56 households per facility, or 28 per sampled EA (total of 838 households in each arm). We also assumed that care-seeking and treatment for children under 5 years of age with pneumonia and diarrhea would not be affected by the intervention.

### Intervention implementation

The intervention was initially planned to begin in March/April 2020 with a duration of 20 months but was delayed for eight months as a result of the COVID-19 pandemic, thus implementation lasted only 14 months. All CHWs in the study area received a refresher training on mCCM for CU5 and data collection tools, and a community sensitization campaign was conducted in October 2020. In addition, CHWs in the intervention arm were trained on age-expanded mCCM. An initial set of supplies was provided to all CHWs at the end of the training, including malaria rapid diagnostic tests (RDTs) and artemisinin-based combination therapy (ACTs), surgical masks for COVID-19 protection, and supplies for waste management. In each commune (grouping of several *fokontany*) in the intervention arm, sensitization on mCCM for all ages was provided to local leaders, including village leaders, mayors, heads of *fokontany*, midwives, and CHWs. Mass gatherings were avoided due to the risk of COVID-19 transmission. In addition, radio broadcasts sensitized the entire study population and included reminders that supplies for malaria diagnosis and treatment were free of charge as part of the current NMCP policy.

The mCCM intervention was implemented from November 1st, 2020, to December 31st, 2021. In both arms, CU5 attending a CHW for febrile illness were managed according to existing iCCM protocols in Madagascar. In brief, CHWs are trained to do an RDT for all CU5 presenting with fever. Children under 2 months of age, CU5 with signs of severe illness (e.g., lethargy, seizures, or inability to breastfeed) are referred to health facilities. Uncomplicated malaria cases should receive artesunate–amodiaquine. In addition, CHWs were instructed to conduct a malaria RDT for all people aged 5 years or older with fever in the intervention arm. RDT-positive individuals were assessed and classified as uncomplicated or severe malaria cases according to national guidelines. All women aged 15–45 years with a positive RDT were asked about their pregnancy status; if women were pregnant or did not know their pregnancy status, they were referred to a health facility in the event of a positive malaria RDT. Individuals presenting with signs of severe illness (e.g., lethargy, seizures, anemia, stroke, abnormal bleeding) were also referred to health facilities regardless of RDT results. All individuals diagnosed with uncomplicated malaria (i.e., RDT-positive, non-pregnant individuals not displaying warning signs) received artesunate–amodiaquine from the CHW. Those with a negative RDT (and CU5 with no other obvious cause of fever according to iCCM) were referred to the nearest health facility.

In both arms, routine monthly reviews with CHWs were conducted at the health center as per standard CHW policy and were supported by a team of study supervisors in partnership with staff from Inter Aide during intervention implementation. The goal of these reviews was to (i) address challenges in case management and the use of different data collection tools by CHWs, providing additional on-site training where necessary, (ii) collect data from health center registers and from CHW monthly reports, and (iii) support supply chain management at the community level by helping CHWs with the supply ordering process and provision of additional malaria supplies in urgent cases (stock-out or near stock-out). In addition, separate coaching sessions were conducted with smaller groups of CHWs needing additional help. Finally, given significant malaria supply chain challenges at multiple levels of the health system, the study team worked closely with the district, regional, and national bodies responsible for malaria supply management in addition to providing supply chain support at the community level (in both arms) and additional storage capacity. CHWs in both arms were provided a small monetary incentive for their participation in the study, amounting to about 15 USD per CHW every 6 months. More details on health system strengthening support in both arms during age-expanded mCCM are available in the Additional file 1: Table S2.

### Data collection

#### Survey data

Two cross-sectional surveys representative of the study area population were conducted prior to (baseline) and after (endline) implementation of the age-expanded mCCM intervention. A random sample of 1680 households without replacement was selected using a two-stage cluster sampling scheme. Prior to the baseline survey, the study area was mapped onto a spatial grid with 2 × 2-km (km) tiles, and two tiles were randomly selected within each health center catchment to serve as enumeration areas (EAs). Sixty EAs were selected, two per health center. Structures mapped through satellite imagery prior to fieldwork were visited by the enumeration team to determine which structures represented inhabited households, and simple random selection was used to select 28 households from each EA. The same EAs were used in both the baseline and endline surveys; baseline and endline sampling of households within each EA were done independently. More details on the study survey design are available at [25].

The baseline cross-sectional survey was conducted between October and December 2019. Interviews were conducted with an eligible household respondent (18 years or older and usual resident of the household) of sampled households. Data collected included a listing of household members; basic socio-economic and demographic information; history of illness in the previous 2 weeks among all household members; care-seeking behaviors, diagnosis, and treatment received for common illnesses (including fever, cough, and diarrhea) and associated costs among all household members; and perceptions of treatment from CHWs and health facilities. In addition, a capillary blood specimen was collected from children aged 2 months to 14 years by finger prick. Blood specimens were used to perform a malaria RDT at the site of collection. Children with a positive RDT were treated with artesunate-amodiaquine and paracetamol. Adults provided written informed consent for the household interview. For the capillary blood collection, parents or guardians provided written consent for children under 15 years of age, and children 7–14 years also provided written assent.

The endline survey was conducted between October and December 2021 in the same EAs. Twenty-eight households were sampled from the baseline sampling frame, and a list of ten replacement households was also generated to account for selected households that had moved since the baseline listing. Households refusing to participate or those absent during three attempted visits by survey teams were not replaced. All other protocols for data collection were the same as during baseline surveys. In addition to the cross-sectional surveys, qualitative data collection was conducted at the endline to gain in-depth knowledge on the acceptability of mCCM, as well as knowledge, attitudes, and practices towards malaria in the study area. Qualitative data and results are not included here.

#### Health system information

Consultation data at the CHW and health center levels were collected from January 2019 to December 2021, including the number of consultations, patients with fever, RDTs done, RDT-confirmed malaria cases, and ACT treatments delivered per month for each *fokontany*. At the CHW level, these data were retrieved from CHW registers and aggregated monthly. At health facilities, patient-level data were retrieved retrospectively from each health facility register prior to study start, and prospectively every month. Registers were photographed and data were entered into a patient-level, de-identified database. Health center data included new visits only and key information abstracted included demographics, patient village or *fokontany* of residence, illness, malaria diagnosis, and treatment. Overall, 8576 months of CHW data out of 9,036 expected (95.0%), and 1055 months of health center data out of 1080 expected (97.7%) were collected. Population data for each *fokontany* were obtained from the Ministry of Public Health and were calculated by applying a constant population growth estimate of 2.7% per year to data collected in the 2018 national census [23]. Using the total population of each *fokontany*, the populations of different age groups were estimated using population structure in our household surveys, where 22.7% were children 0–5 years, 26.1% were children 6–13 years, and 51.2% were individuals 14 + years old.

To estimate the average distance of a *fokontany’s* population to the nearest health center, we built on work developed by Ihantamalala et al. [26]. Briefly, all footpaths, residential areas, and buildings in the district were mapped between July 2021 and October 2022 using very high-resolution satellite images available through OpenStreetMap (OSM), resulting in 174,675 buildings, 11,592 residential areas, 628 km of non-paved roads and 27,699 km of footpaths mapped. When mapping was completed, the Open Source Routing Machine (OSRM) engine was used to query OSM data and estimate the shortest path between each building in the district and the nearest health center. The aggregated health center distance for a *fokontany* was the average distance from all buildings in the *fokontany*.

### Data analysis

#### Analysis of survey data

Descriptive analyses of individual and household characteristics for the baseline and endline surveys were performed. To estimate the impact of age-expanded mCCM, the proportion of individuals who sought care at a public health provider among those with a fever in the previous 2 weeks, the proportion receiving an RDT, and the proportion of RDT-positive cases receiving an antimalarial treatment were estimated for the intervention and control areas. Difference-in-differences (DiD) analyses were conducted via multivariate logistic regressions to evaluate the impact of age-expanded mCCM while controlling for differences between study periods (endline vs. baseline) and between the two arms (intervention vs. control). Analyses were done by type of care accessed (health facility, CHW, or both), by reported travel time to a health facility (< 1 h, 1–2 h, > 2 h), and by age group (0–5 years, 6–13 years, 14 + years). These age groups were selected instead of the initially pre-specified groups (under 5 years, 5–14 years, 15 + years) to correspond to categories in CHW reports used in health system analyses (next section), which were based on age groups for ACT medications used in Madagascar. Sampling weights adjusting for unequal probabilities of selection were calculated for each household. All estimates used applicable design weights and survey commands available in the R package *survey* [27].

#### Analysis of routine health information system data

Data collected from health centers and CHWs included key indicators (e.g., numbers of consultations, fever cases, RDTs, RDT-confirmed malaria cases, and antimalarial treatments) and were aggregated by *fokontany*, month, and age group of patients. Together, health center and CHW datasets allowed us to obtain precise estimates of the spatio-temporal evolution of health-seeking behaviors, malaria diagnosis, and case management at the primary care level (health centers and CHWs).

The impact of the intervention on key indicators was modeled using interrupted time-series analyses, with *fokontany* as the unit of analysis. Negative binomial regressions were used in generalized additive mixed models, with a random intercept for the *fokontany* and the logarithm of the *fokontany* population as offset. Outcome variables included the number of consultations with febrile illness, RDTs done, and antimalarial treatments delivered, both at health center and CHW levels. For each model, the intervention impact was estimated by assessing both the level of change and the slope of change associated with it, controlling for differences between study arms (intervention vs. control) and periods (after vs. before the intervention began). The level of change is the interaction between study arms and periods and represents the average change associated with the intervention, equivalent to a difference-in-differences estimator. The slope of change is the interaction between the level of change and time and represents the change in intervention impact over time after accounting for its average impact. The interaction between the intervention level of change and the average distance from the fokontany to the nearest health center was studied to assess whether the impact was different for more remote populations. The analysis controlled for temporal trends in utilization rates during the study period, including linear (i.e., time since January 2019), seasonal (*sine* function), and lagged (1-month lag) trends. It also controlled for the non-linear effect of distance from a *fokontany* to the nearest health center using a cubic regression spline. Consistent with survey analyses, healthcare facility, and CHW data consultations were first modeled together and then separately, and separate models were carried out for each age group. Supplementary analyses were carried out to assess the impact of the intervention on the rates of acute respiratory infections (ARI) and diarrhea cases among children under 5 years seen at the CHW level, as well as treatment rates. Moreover, in order to understand whether the COVID-19 pandemic was responsible for the increase in fever care-seeking cases, we assessed the evolution of malaria RDT positivity at both levels of care, assuming that an increase in COVID-19 fevers would result in a decrease in overall malaria RDT positivity during the 2020 and 2021 waves. All analyses were done using R software version 4.2.1 and time-series analyses were done using R package *mgvc* [28].

## Results

### Population characteristics

The study population comprised over 350,000 people evenly distributed into the two study arms (Table 1). Children under 14 years represented half of the population, and nearly one in four people lived further than 5 km from the nearest health center. From January 2019 to December 2021, 462,215 consultations and 382,187 RDTs were done in the 30 health centers in the study area. Similar numbers (464,440 consultations and 370,267 RDTs) were done by the 502 CHWs. The numbers of RDTs done and ACTs administered were similar for health facilities in both arms but were higher for CHWs in the intervention arm. For populations living further than 5 km from a health center, the number of consultations at health facilities was substantially lower than at CHWs (Table 1).

**Table 1:** Population and health system characteristics in the study area, 2019–2021

	**Study area**	**Study arm**		**Distance to health center**	
		Control	Intervention	Less than 5 km	5 km or more
**Population (2019)**^**a**^					
All ages	363,962	177,858	186,104	228,340	135,622
0–5 years	82,617	40,372	42,245	51,831	30,786
6–13 years	94,998	46,421	48,577	59,601	35,397
14+ years	186,347	91,065	95,282	116,908	69,439
**Health center level (01/2019–12/2021)**^**b**^					
Number of facilities	30	15	15	-	-
Consultations	462,215	239,679	222,536	363,729	98,486
Fever cases	307,752	160,367	147,385	240,069	67,683
RDTs done	382,187	193,294	188,893	301,226	80,961
Malaria cases (RDT+)	221,845	111,791	110,054	175,534	46,311
ACTs administered	217,202	108,708	108,494	171,913	45,289
**Community health level (01/2019–12/2021)**^**c**^					
Number of CHWs	502	262	240	296	206
Consultations	464,440	201,555	262,885	275,196	189,244
Fever cases	422,271	177,709	244,562	251,874	170,397
RDTs done	370,267	154,785	215,482	222,902	147,365
Malaria cases (RDT+)	242,682	94,436	148,246	144,095	98,587
ACTs administered	228,304	87,282	141,022	134,722	93,582

^a^Source: MoPH sectorization, based on 2018 national census

^b^Source: health center registers

^c^Source: CHW registers and monthly reports

During the baseline and endline household surveys, 1458 (86.8%) and 1631 (97.1%) households participated, respectively (Table 2). The study population had low levels of basic education, was primarily agricultural, and had largely low socio-economic levels (e.g., lacked electricity or toilets). Households were located over two times farther from health centers on average (over 1 h walking) than from CHWs (less than 30 min). The proportion of households with one or more members who sought care for any reason at the CHW increased during the study period (from 73.9 to 81.4%) and decreased at health centers (from 83.2 to 73.3%). Fever was the main reason for seeking care, but the proportion was much larger at the CHW level (baseline: 82.4%, endline: 91.8%) than at health centers (baseline: 52.1%, endline: 57.7%). Of 8,050 individuals of all ages listed at baseline and 9046 at endline, only 6.2% and 4.7% reported being ill in the previous 2 weeks, respectively. RDT prevalence of malaria in children under 15 years increased from 22.4 to 27.1% from baseline to endline (Table 2). Children 5–14 years had about twice the malaria prevalence as CU5 in both surveys but were less likely to report recent fever.

**Table 2:** Characteristics of households and individuals participating in the baseline and endline surveys in Farafangana, Madagascar

**Variable**	**Category**	**Baseline**	**Endline**
		**Average (95% CI)**	**Average (95% CI)**
**Households characteristics — socio-demographics**		***N* = 1458**	***N* = 1631**
Age of head of household in years, mean		41.5 (40.0–43.0)	43.9 (42.3–45.4)
Male head of household, %		58.2 (52.0–64.2)	68.5 (64.7–72.0)
No primary education of head of household, %		84.6 (80.1–88.2)	81.3 (76.1–85.6)
Occupation of head of household occupation, %	Farming or agriculture	76.4 (66.9–83.9)	85.9 (78.7–90.9)
	Day laborer	11.5 (7.7–16.9)	4.9 (2.7–8.9)
	Other	12.1 (7.5–18.9)	9.2 (6.3–13.4)
No toilet in the home, %		68.6 (58.8–77.0)	58.4 (48.8–67.5)
No electricity in the home, %		94.5 (92.0–96.3)	94.9 (90.0–97.4)
Number of bed nets used per sleeping space in household, %		0.95 (0.92–0.98)	0.97 (0.96–0.98)
**Household characteristics — healthcare access and use**			
Time to CHW, one-way, mean hours		0.5 (0.42–0.58)	0.45 (0.39–0.52)
Last visit to CHW, %	≤ 1 year	75.9 (69.4–81.4)	81.3 (77.7–84.5)
Any household member	> 1 year	8.1 (6.2–10.6)	11.5 (9.3–14.1)
	Never	16.0 (10.8–23.0)	7.2 (5.2–9.9)
Reason for last visit to CHW, %	Fever	83.3 (79.0–86.8)	91.8 (89.3–93.8)
Any household member	Cough	8.2 (6.3–10.6)	4.2 (2.9–6.2)
	Diarrhea	2.2 (1.3–3.7)	1.2 (0.6–2.4)
	Other	6.4 (4.3–9.4)	2.7 (1.8–4.1)
Time to the health center, one way, mean hours		1.48 (1.13–1.82)	1.54 (1.24–1.9)
Last visit to the health center, %	≤ 1 year	81.5 (77.2–85.2)	70.1 (65.3–74.5)
Any household member	> 1 year	11.6 (9.2–14.5)	19.9 (16.7–23.6)
	Never	6.9 (4.7–10)	10 (6.8–14.4)
Reason for the last visit to the health center, %	Fever	52.2 (46.7–57.6)	58.7 (54.4–62.8)
Any household member	Cough	10.7 (8.3–13.8)	10.1 (7.5–13.3)
	Diarrhea	5.9 (4.5–7.6)	3.6 (2.3–5.7)
	Other	31.2 (26.8–36)	27.7 (23.9–31.8)
**Individual characteristics**		***N* = 8050**	***N* = 9046**
Sex, %	Male	47.7 (46.6–48.9)	48.5 (47.1–49.9)
Age, %	< 5 years	19.4 (18.5–20.3)	19.1 (18–20.2)
	5–14 years	30 (28.4–31.7)	28.5 (27.4–29.7)
	15 + years	50.6 (49.1–52)	52.4 (50.8–54)
Individual ill in previous 2 weeks, %		6.7 (5.3–8.4)	5.1 (4–6.6)
Febrile illness, %		82.2 (76.6–86.8)	74.7 (67.6–80.7)
Care-seeking for fever at HF or CHW, %		22.8 (16.5–30.6)	60.7 (49.3–71)
mRDT done for fever at HF or CHW, %		20.0 (14.2–27.4)	59.3 (48.7–69.1)
ACT treatment provided for mRDT + , %		87.7 (72.2–95.1)	95.5 (91.4–97.7)
Diarrhea (children under 5 years), %		0.6 (0.3–1.4)	1.6 (0.9–2.8)
Pneumonia (children under 5 years), %		3.9 (2.8–5.4)	5.1 (3.1–8.3)
Care-seeking for pneumonia at HF or CHW, %		22.2 (11–39.7)	50.4 (38–62.7)
Treatment for pneumonia at HF or CHW, %		16.1 (7.3–31.7)	22 (9.9–42)
**Malaria prevalence — children under 15 years**		***N* = 3316**	***N* = 3905**
Malaria prevalence (mRDT+, %)		25 (19.4–31.2)	30.4 (24.9–36.2)
mRDT+ with recent fever (last 2 weeks, %)		21.5 (15.6–28.5)	10.1 (7.7–13)
Prevalence by sex (mRDT+, %)	Female	23.3 (17.5–29.9)	26.8 (21.3–32.9)
	Male	26.8 (20.8–33.5)	33.7 (28.0–39.7)
Prevalence by age group (mRDT+, %)	Under 5 years	15.7 (10.1–22.9)	19.5 (15.4–24.1)
	With recent fever	26.6 (15.2–41.0)	17.5 (12.3–23.8)
	5–14 years	31.5 (25.4–38.2)	37.6 (30.9–44.7)
	With recent fever	19.7 (13.9–26.7)	7.5 (5.2–10.5)

### Impact of age-expanded mCCM

**Fig. 2:**
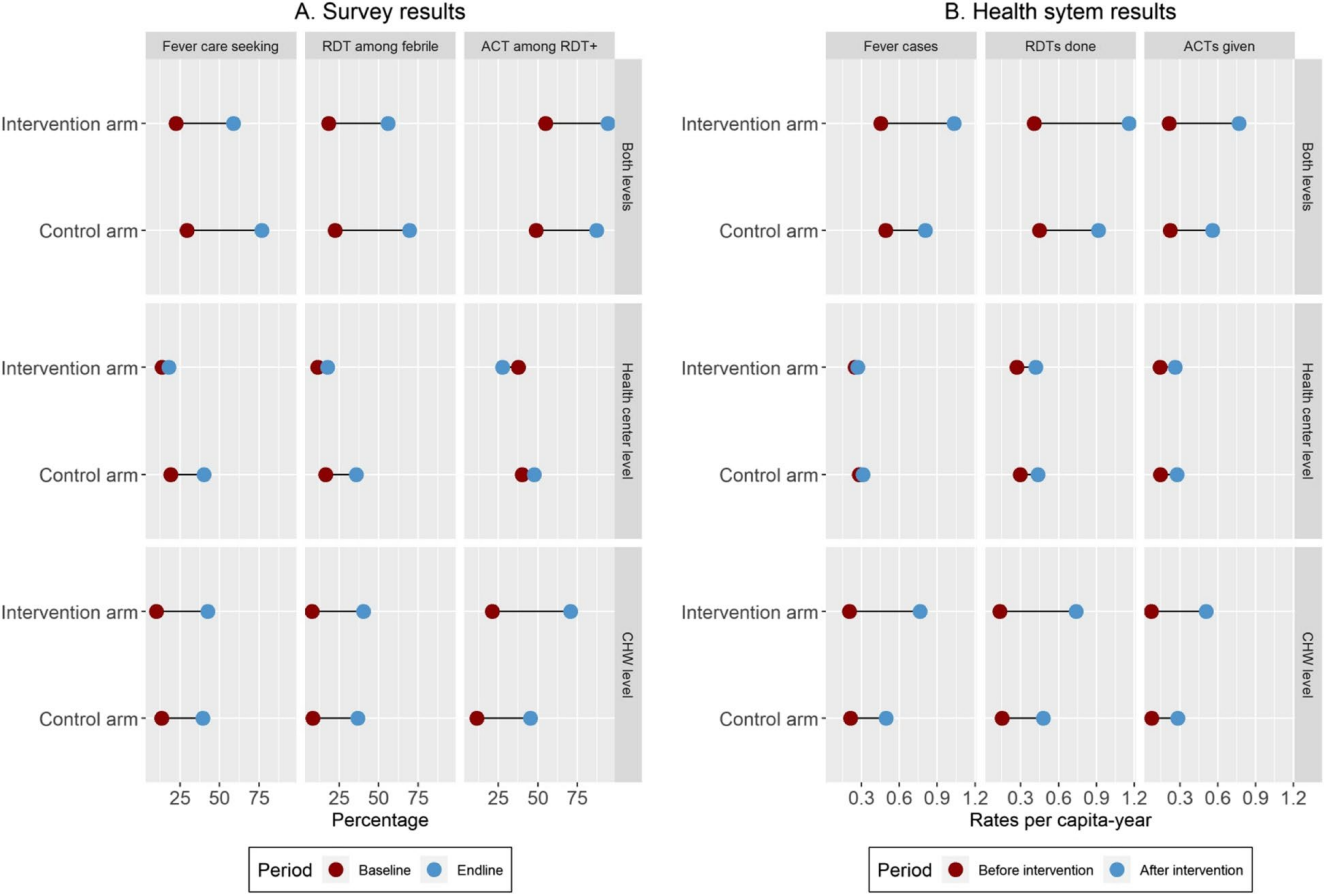
Average changes in key malaria indicators before and after mCCM implementation in each study arm. **A** Results from household surveys, estimated among all individuals who reported a fever (for care-seeking and RDT diagnosis) or an RDT+ (for ACT) in the 2 weeks prior to the survey. **B** Results from health system information, estimated from monthly primary care consultations at health centers and CHWs among the total population in the study area. Results for both levels of care (top panels) in both analyses (survey, health system) represent the sum of percentages or rates from each level of care (health center and CHW level)

Rates of care-seeking for fever, malaria diagnosis, and treatment substantially increased across the study area after implementation of the intervention (Table 2). Of 717 individuals who reported a fever in the 2 weeks prior to the survey, the weighted average of care-seeking and malaria RDT diagnosis more than doubled in both arms from less than 25% at baseline to over 60% at endline (Table 2 and Fig. 2A). Similarly, the percentage of ACT treatments provided by health facilities or CHWs among survey individuals who reported having an RDT + diagnosis doubled from 50% to nearly 100%. In the control arm, these improvements were driven equally by increases at the health center and at the CHW level, whereas in the intervention arm, they were mostly driven by increases at the CHW level (Fig. 2A). Results from difference-in-difference analyses found that age-expanded mCCM was associated with an increase in malaria diagnoses at the CHW level and a decrease at the health center level, but none of these effects were significant (Table 3). Malaria prevalence slightly increased between baseline and endline, although the proportion of symptomatic malaria declined, especially in children 5–14 years old (Table 2).

**Table 3:** Impact of age-expanded mCCM on the proportion of RDTs done among febrile individuals^a^ (logistic regression, difference-in-differences analyses using survey data)

**Age group**	**Level of care**	**Intercept**	**Change over time (ref. baseline)**	**Arm differences (ref. control arm)**	**Difference-in-differences (Period × Arm)**	**Observations**
			**OR (95% CI)**	**OR (95% CI)**	**OR (95% CI)**	
All ages	Both levels of care	0.22 (0.14–0.33)***	8.46 (3.72–19.23)***	0.88 (0.37–2.1)	0.76 (0.24–2.38)	717
	Health center	0.15 (0.09–0.25)***	3.25 (1.09–9.71)*	0.88 (0.25–3.06)	0.55 (0.12–2.57)	717
	CHW	0.09 (0.04–0.18)***	6.21 (2.25–17.17)***	0.83 (0.28–2.43)	1.46 (0.43–4.97)	717
Children 0–5 years	Both levels of care	0.24 (0.13–0.4)**	20.53 (6.74–62.51)***	0.79 (0.31–2.03)	0.4 (0.09–1.86)	261
	Health center	0.18 (0.08–0.33)***	2.77 (0.59–12.95)	0.49 (0.17–1.43)	1.1 (0.18–6.71)	261
	CHW	0.08 (0.03–0.19)***	11.73 (3.44–40.03)***	1.43 (0.37–5.48)	0.63 (0.12–3.18)	261
Children 6–13 years	Both levels of care	0.24 (0.13–0.41)**	10.03 (2.5–40.16)**	0.73 (0.22–2.44)	0.75 (0.12–4.65)	229
	Health center	0.13 (0.07–0.24)***	6.63 (1.76–24.94)**	0.97 (0.2–4.62)	0.21 (0.03–1.69)	229
	CHW	0.13 (0.05–0.31)**	2.61 (0.75–9.16)	0.5 (0.12–2.04)	4.38 (0.81–23.64)	229
Individuals 14+ years	Both levels of care	0.17 (0.09–0.31)***	2.74 (0.84–8.98)	1.27 (0.4–4.04)	1.51 (0.29–7.92)	226
	Health center	0.13 (0.07–0.24)***	1.39 (0.51–3.8)	1.38 (0.38–5.04)	0.99 (0.2–4.96)	226
	CHW	0.05 (0.01–0.18)***	6.61 (1–43.85)	0.8 (0.12–5.18)	1.53 (0.15–16.11)	226

^*^p < 0.05; **p < 0.01; ***p < 0.001

^a^Equivalent results for fever care-seeking and malaria ACT treatments are available in Additional file 1

In analyses of health system data, which included information from the 926,655 primary care consultations occurring between January 2019 and December 2021, rates of fever care-seeking (consultations) and malaria diagnosis and treatment increased in both intervention and control arms, but these increases were smaller in the control arm (Fig. 2B). For instance, the annual number of per capita RDTs performed in the intervention arm increased from 0.45 before the intervention to 1.16 after the intervention was implemented, and from 0.44 to 0.91 in the control arm. Similar to survey analyses, consultation increases in the intervention arm were driven by large increases at the CHW level. In contrast with survey analyses, increases at the health center level in the control arm were very small. Interrupted time-series analyses revealed that when considering both levels of care, age-expanded mCCM was not associated with a significant increase in malaria diagnoses for people of all ages across the intervention area (RR_level_ = 0.96; 95% CI 0.88–1.06), but was associated with a significant increase in malaria diagnosis for populations living further from health centers (RR_km_ = 1.08; 95% CI 1.06–1.09), particularly among individuals older than 5 years. Moreover, age-expanded mCCM was associated with a significant increase in malaria diagnosis at the CHW level both for the level of change (RR_level_ = 1.28; 95% CI 1.08–1.5) and the slope of change (RR_slope_ = 1.30; 95% CI 1.12–1.51).

**Fig. 3:**
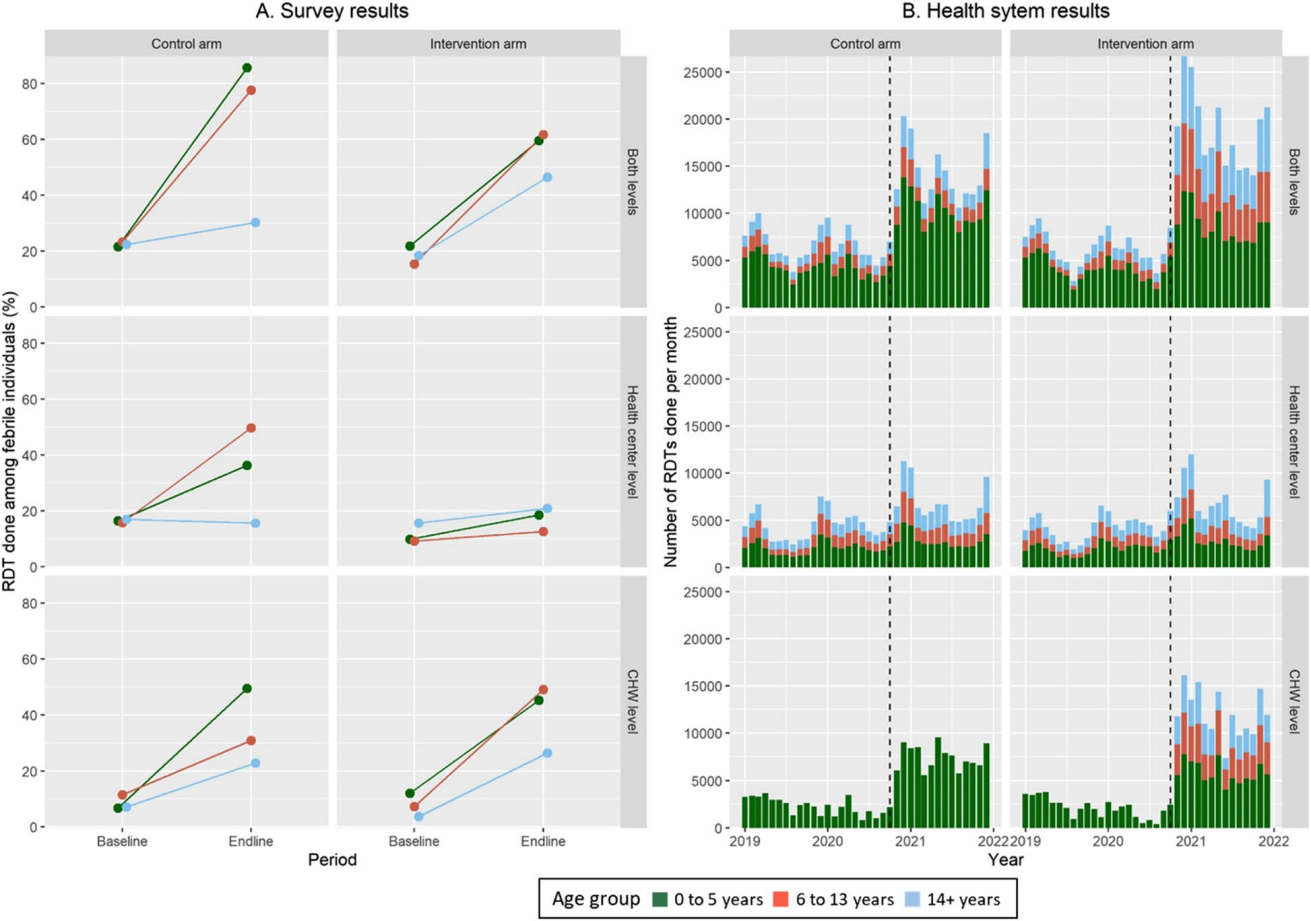
Changes in rates of malaria diagnosis (RDTs) by age group before and after mCCM implementation in each study arm. **A** Results from household surveys, comprising individuals who reported a fever in the 2 weeks prior to the survey. **B** Results from health system information, comprising monthly primary care consultations at health centers and CHWs. Dotted vertical line indicates the beginning of HSS support and age-expanded mCCM. Equivalent figures for fever care-seeking and malaria treatments are available in the Additional file 1

When considering specific age groups, rates of malaria testing at both care levels increased particularly for children 0–5 years and 6–13 years, tripling in both arms according to survey analyses, while they doubled for individuals 14 + years (Fig. 3A). About 50% of all children 0–5 and 6–13 years in the survey who reported a recent fever in the intervention arm were tested at the CHW level at endline, from levels around 10% at baseline, while individuals 14 + years experienced a modest increase of about 20 percentage points (Fig. 3A). Analyses of health system data revealed that these improvements occurred immediately after intervention implementation and were sustained throughout the study period (Fig. 3B). Malaria diagnosis for individuals older than 5 years also occurred before age-expanded mCCM and rates increased substantially at the CHW level in the control arm according to survey data (Fig. 3A), even though age-expanded mCCM was not officially in place, and therefore, this was not reported in health system data (Fig. 3B). None of the difference-in-difference results from survey data was significant for malaria diagnosis for specific age groups (Table 3, Table S3). In contrast, results from interrupted time-series analyses (Table 4) revealed that age-expanded mCCM was associated with significant increases in the level of change for malaria diagnosis children 6–13 years (RR_level_ = 1.65; 95%CI 1.45–1.87) and for individuals 14 + years (RR_level_ = 1.46; 95%CI 1.3–1.63), although this effect decreased slightly over time (RR_slope_ = 0.88 and RR_slope_ = 0.87 per year, respectively). For CU5, large improvements were seen in both arms, but the improvement was larger in the control arm (Table 4), reflected in an RR_level_ of 0.76 (95%CI 0.68–0.84) for age-expanded mCCM on the rates of malaria diagnosis in this age group. Similar results were observed in analyses of rates of fever consultations and ACT treatments (Additional file 1: Table S4). The time-series models using health system data predicted well the spatio-temporal trends observed in the data and explained about 40–70% of the variance in the data (Additional file 1: Figures S1–S2).

**Table 4:** Impact of age-expanded mCCM on the number of RDTs done per month^a^ (negative binomial regression, interrupted time-series analyses^b^ using health system data)

**Age group**	**Level of care**	**Change over time (ref. before)**	**Arm differences (ref. control)**	**Impact of mCCM (level of change)**	**Impact of mCCM (slope of change)**	**Impact of mCCM over distance to HF (km)**
		**RR (95% CI)**	**RR (95% CI)**	**RR (95% CI)**	**RR (95% CI)**	**RR (95% CI)**
All ages	Both levels of care	2.3 (2.17–2.44)***	0.93 (0.8–1.07)	0.96 (0.88–1.06)	0.99 (0.91–1.07)	1.08 (1.06–1.09)***
	Health center	1.11 (1.05–1.18)***	0.97 (0.77–1.23)	1.08 (0.98–1.2)	0.79 (0.72–0.87)***	1.01 (1–1.03)
	CHW	5.6 (5.04–6.21)***	0.95 (0.84–1.09)	1.28 (1.08–1.5)**	1.30 (1.12–1.51)***	1.02 (1–1.04)
Children 0–5 years	Both levels of care	2.7 (2.53–2.88)***	0.92 (0.8–1.06)	0.76 (0.68–0.84)***	1.01 (0.92–1.11)	1.04 (1.03–1.05)***
Children 6–13 years	Both levels of care	1.2 (1.11–1.29)***	1.15 (0.92–1.44)	1.65 (1.45–1.87)***	0.88 (0.79–0.98)*	1.21 (1.19–1.23)***
Individuals 14+ years	Both levels of care	1.32 (1.23–1.41)***	1.12 (0.91–1.38)	1.46 (1.3–1.63)***	0.87 (0.79–0.95)**	1.18 (1.16–1.19)***

^*^p < 0.05; **p < 0.01; ***p < 0.001

^a^Equivalent results for the number of fever cases and malaria ACT treatments given are available in the Additional file 1

^b^Analyses were controlled for linear time trends, seasonality, lagged utilization (t-1 month), and a non-linear smooth for distance from fokontany to nearest health center

**Fig. 4:**
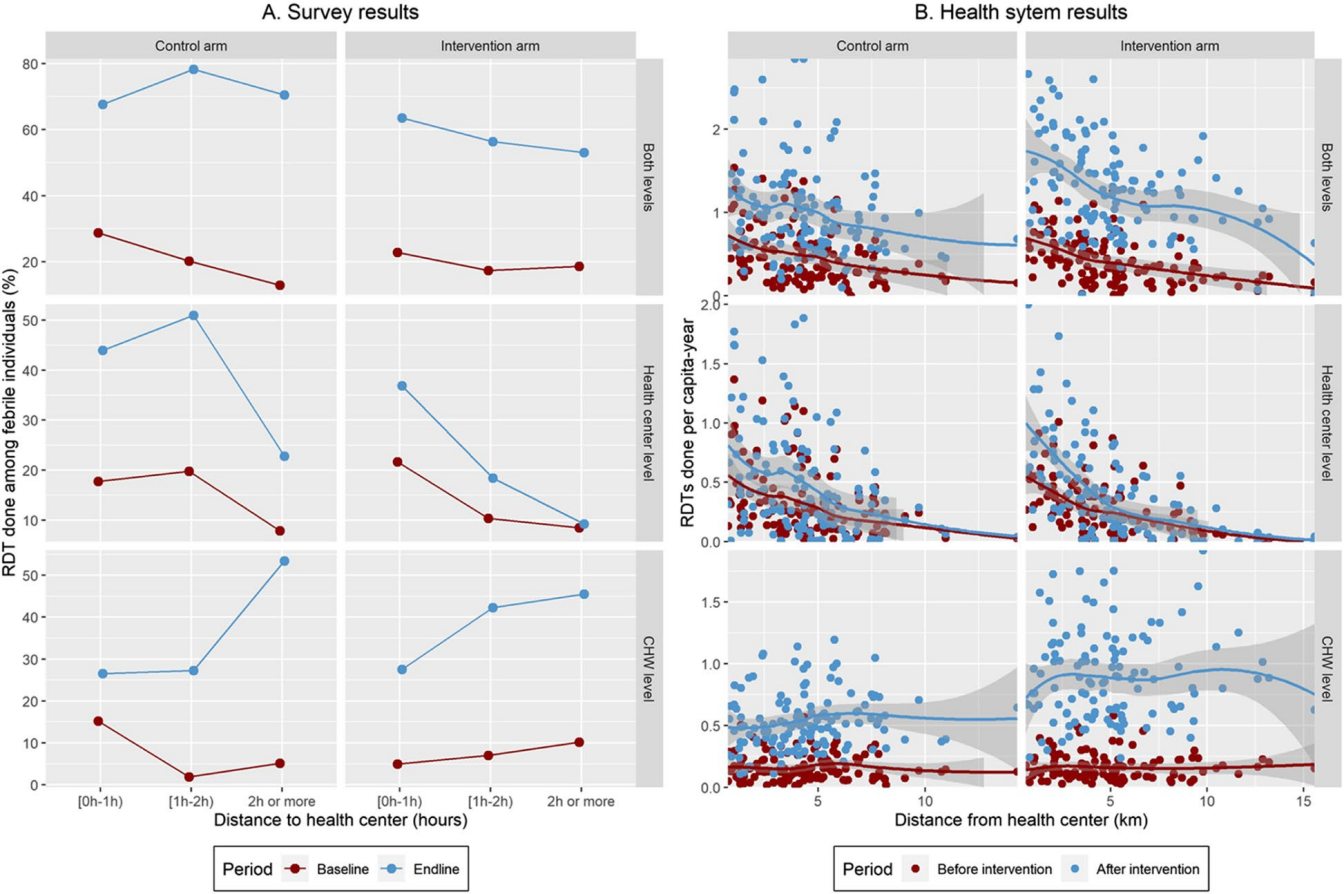
Changes in rates of malaria diagnosis (RDTs) by population distance to health centers before and after mCCM implementation in each study arm. **A** Results from household surveys, comprising individuals who declared being ill in the previous 2 weeks and reported travel time to the nearest health center. **B** Results from health system information, comprising monthly primary care consultations at health centers and CHWs and estimated distance to the nearest health center via OSRM. Each dot represents the average of one *fokontany*, with solid lines representing the fitted smooth from a general additive model and its 95% confidence intervals (gray area). Note that *y*-axis scales are different. Equivalent figures for fever care-seeking and malaria treatments are available in the Additional file 1

Analyses of geographic inequalities revealed that populations living closer to a health center had higher overall rates of malaria diagnosis (Fig. 4). There was an exponential distance decay in the rates of malaria diagnosis at the health center level, which was more pronounced in the intervention arm and which was exacerbated after intervention implementation. In contrast, rates of malaria diagnosis by CHWs, which were low in both arms pre-intervention, increased for all populations regardless of their distance to a health center (Fig. 4). The effect was larger in the intervention arm, where malaria diagnosis reached an average of nearly 1 RDT per person per year for nearly all distance groups, versus less than 0.5 RDT per person per year in the control arm (Fig. 4B). Results from interrupted time-series analyses (Table 4) revealed that the intervention impact was significantly larger overall for every additional km that populations lived from the health center (RR_km_ = 1.08; 95% CI 1.06–1.09), and this effect was particularly high for children 6–13 years (RR_km_ = 1.21; 95% CI 1.19–1.23) and individuals 14 + years (RR_km_ = 1.18; 95% CI 1.16–1.19).

The dramatic increases in the rates of care-seeking for fever observed during the study period seemed unrelated to the COVID-19 pandemic. We observed that there was a sudden and sustained increase in care-seeking at the community level (as well as in malaria RDTs done and treatments given) coinciding with the beginning of intervention implementation in November 2020 (Fig. 3, Additional file 1: Figures S3–S5), while the first COVID-19 waves in Madagascar occurred in July–August 2020 and in April–May 2021. Moreover, malaria positivity rates remained fairly stable over the whole time series and even increased after 2019 both at health facilities and at the community level (Additional file 1: Figure S13).

Rates of ARI and diarrhea cases seen at the CHW level among CU5 increased in both arms following the intervention, as well as rates of antibiotic treatments given for ARI and oral rehydration salts given for diarrhea (Additional file 1: Figure S7 and Table S6). However, these increases were significantly smaller in the intervention arm than in the control arm (Table S6). Analysis of stock data gathered from CHWs during the study period revealed that stocks of key malaria commodities increased at both health center and CHW levels, and stock-outs reduced during the intervention period (Additional file 1: Figures S8–S12).

## Discussion

This randomized trial in rural Madagascar assessed how expanding mCCM to all ages impacts access to malaria diagnosis and treatment. Our results demonstrate that age-expanded mCCM led to significant increases in rates of care-seeking for fever, malaria diagnosis, and treatment for individuals over 5 years. The impact of age-expanded mCCM was larger for remote populations, effectively reducing geographic inequalities in the study area. Substantial increases were also observed in the control arm following the CHW program and supply chain enhancements that were done in support of the trial. The increases in fever care-seeking observed did not seem associated with the COVID-19 pandemic. Thus, while expanding mCCM to all ages could facilitate universal access to malaria diagnosis and treatment, strengthening current iCCM programs and malaria supply chains could achieve significant improvements in access to malaria care even in the absence of age-expanded mCCM.

Our study fills a critical gap in the evidence for expanding the role of CHWs in the provision of uncomplicated malaria care to individuals over 5 years. Although retrospective observational evidence suggests that in Rwanda age expansion of mCCM improved access to malaria diagnosis and treatment [17], most countries with high malaria burdens have not yet adopted this approach, which could be due to concerns about feasibility, limited resources, acceptability, and/or impact. In our trial, age-expanded mCCM resulted in an immediate and sustained uptake of this intervention by individuals of all ages, resulting in over 85,000 RDTs done and 60,000 ACT treatments provided for individuals older than 5 years of age at the community level during the 14-month implementation period, equivalent to 1.2 additional RDTs done by CHWs per workday. Overall, annual rates per capita roughly tripled in the intervention arm for RDTs done (from 0.41 to 1.16) and for ACTs given (from 0.21 to 0.77), an increase comparable to that observed in a recent study of proactive (home-based) malaria CCM in south-eastern Madagascar [16]. Survey results revealed that CHW diagnosis and treatment of older ages for fever was already taking place before the intervention started, and this practice increased in the control arm following implementation (Fig. 3). This suggests there is an underlying demand for age-expanded mCCM, especially in remote populations (Fig. 4). This could have limited the impact we observed with age-expanded mCCM, which was smaller than the impact associated with strengthening the CHW and supply chain systems in both arms and was not statistically significant in survey analyses. Despite this, analyses of health system data show that age-expanded mCCM was independently associated with an overall increase of about 50% in the rates of malaria diagnosis and treatment for individuals over 5 years (RR_level_ = 1.65 for individuals 6–13 years and RR_level_ = 1.46 for individuals 14 + years; Table 4). The decrease in symptomatic parasitemia seen between the baseline and endline surveys might be due to increased rates of care-seeking for febrile illness among older children.

The goal of community health programs is to increase access to quality care for populations who live far from health centers. Health system data revealed an exponential decrease in health center utilization the farther people lived from a health center. However, CHW utilization remained stable or even increased with populations’ distance to health centers because the CHWs were embedded in their communities (Fig. 4). As a result, age-expanded mCCM was associated with an additional relative increase of about 20% in rates of malaria diagnosis among individuals older than 5 years for every extra kilometer that they had to travel to the nearest health center (Table 4). These results are consistent with a previous study in rural Madagascar, which showed that community health programs largely compensated for the distance decay in health center utilization and reduced geographic inequalities in access to primary health care [29]. Using an ingredients-based costing approach to evaluate the budgetary impact of implementing the age-expanded mCCM program in Farafangana district from a health system perspective (Additional file 2: Table S7), we estimated the total cost of running the expanded program at $794,270 per year in the district’s study area, which translates to approximately $2.55 per suspected case of uncomplicated malaria diagnosed and treated in the community (Additional file 2: Table S8). Drugs and consumables accounted for 94% of the cost, and the estimate included initial costs of training and sensitization, as well as capital and supervision costs of the strengthened system (e.g., purchase of a vehicle and motorcycles) but not research-related costs. This represents less than half the cost of outpatient care of uncomplicated malaria cases at health facilities estimated in other settings [30].

The largest observed impact on care-seeking and malaria case management in our study occurred in both arms and was attributed to strengthening community health systems in a setting with low baseline access to care. Prior to the mCCM study, limited stocks of malaria supplies were distributed at the community level, which resulted in frequent stock-outs (Additional file 1: Figure S12). By providing strong support to the malaria supply chain during the intervention (at national, district, and community levels; Additional file 1: Table S2), monetary incentives to CHWs, and widespread sensitization on the availability of free malaria care, our study may have simultaneously increased demand while allowing CHWs to provide more services given increased supplies. For instance, the proportion of people who paid for malaria care among those who sought care from a CHW decreased from 93 to 60% (Additional file 1: Table S5). In addition, qualitative research (data not shown) indicated that local populations began to appreciate that malaria could affect individuals over 5 years of age, and communities put pressure on CHWs and health center staff to conduct RDTs upon consultation. As a result of these mCCM implementation strategies, rates of malaria diagnosis for individuals of all ages more than doubled according to health system data (RR = 2.3), and this effect was even larger (OR = 8.5) in survey data. Improvements in malaria care at the CHW level did not result in a worsening of care for acute respiratory infections or diarrhea among children under 5 (both of which are part of standard iCCM), although the increases seen in the rates of diagnosis and treatment for these two diseases after the intervention was implemented were smaller in the intervention arm than in the control arm (Additional file 1: Figure S7 and Table S6).

Our study had several limitations. First, we powered the household survey assuming a 15% prevalence of reported fever in the previous 2 weeks, in accordance with previous studies in south-eastern Madagascar [31], but reported fever was much lower at under 5% in both baseline and endline surveys. This resulted in very large uncertainty in our estimates and no statistically significant impact was observed for the age-expanded mCCM in survey analyses. However, survey results were consistent with those observed in our analyses of nearly one million primary care consultations at health centers and CHWs (Figs. 2, 3, and 4), which found the impact of age-expanded mCCM to be statistically significant (Table 4). Second, the intervention was initially planned to be implemented for nearly 2 years, but the COVID-19 epidemic shortened the duration of intervention to 14 months. This could have affected our ability to detect medium-term changes in intervention uptake over time. Moreover, the COVID-19 pandemic could have resulted in an increase number of non-malaria febrile illness and consultation rates. However, consultation rates remained stable during the first 8 months of the Madagascar COVID-19 epidemic and only increased upon beginning the intervention in November 2020 (Fig. 3), which suggests the pandemic had little effect on the results observed. Moreover, malaria positivity rates remained stable throughout the study period (Additional file 1: Figure S13). Third, we strengthened the malaria supply chain to limit stock-outs (Additional file 1: Table S2) during study implementation, since this is known to be an obstacle for delivery of mCCM. This, among other health system strengthening activities, had a larger effect than anticipated, resulting in a doubling in the rates of per-capita fever cases presenting for care and RDTs done in the control arm (Fig. 2B). Although this effect is accounted for in our statistical analyses, it is unclear how a setting of frequent stock-outs and lack of external support to community health programs would influence the impact of age-expanded mCCM.

## Conclusion

Our results suggest that an age-expanded mCCM can have a positive impact on access of older individuals to malaria diagnosis and treatment, especially for children 6–13 years of age and for populations living far from health facilities. Moreover, simultaneously strengthening community health activities and supply chains for malaria in rural settings where baseline access to iCCM is low can lead to substantial improvements even in the absence of age-expanded mCCM. A national scale-up of age-expanded mCCM is underway in Madagascar following the results from this pilot study, and a second randomized trial is being conducted in Malawi. Together, these can inform community health policies for malaria control in other sub-Saharan African countries.

### Supplementary Information

Additional file 1: Table S1. Details on primary and secondary outcomes used to evaluate the impact of age-expanded mCCM. Table S2. Health system strengthening activities and solutions implemented in both arms during mCCM expansion period. Table S3. Impact of mCCM expansion to all ages on the proportion of febrile individuals seeking care, and the proportion of RDT+ individuals receiving an ACT. Table S4. Impact of mCCM expansion to all ages on the number of febrile individuals seeking care, and the number of ACTs delivered. Table S5. Reported cost of malaria care at different levels of care for individuals who sought care. Table S6. Impact of mCCM expansion to all ages on the number of children under 5 years seeking care for diarrhea and pneumonia from CHWs. Fig S1. Comparison of observed temporal utilization patterns and multivariate model predictions. Fig S2. Comparison of observed geographic utilization patterns and multivariate model predictions. Fig S3. Changes in rates of fever care seeking by age group before and after mCCM implementation in each study arm. Fig S4. Changes in rates of fever care seeking by population distance to health centers before and after mCCM implementation in each study arm. Fig S5. Changes in rates of ACT treatments by age group before and after mCCM implementation in each study arm. Fig S6. Changes in rates of ACT treatments by population distance to health centers before and after mCCM implementation in each study arm. Fig S7. Changes in rates of ARI and diarrhea case management at community level before and after mCCM implementation in each study arm. Fig S8. RDT stocks at health facility level, before and after mCCM implementation. Fig S9. ACT stocks at health facility level, before and after mCCM implementation. Fig S10. RDT stocks at CHW level, before and after mCCM implementation. Fig S11. ACT stocks at CHW level, before and after mCCM implementation. Fig S12. Monthly stockout days at CHW level for ACTs for children <5 years, before and after mCCM implementation. Fig S13. Evolution of RDT positivity over time in Farafangana District, 2019–2021.

Additional file 2: Supplementary analyses on intervention costing. Table S7. Data Sources for the different categories included in the costing analysis. Table S8. Average costs of diagnosis and treatment of suspected cases of uncomplicated malaria at the community level.

Additional file 3. Consort 2010 checklist.

## Data Availability

Data are available upon request to the address andres.garchitorena@ird.fr.

## Abbreviations

ACT: Artemisinin-based combination therapy
ARI: Acute respiratory infection
CHW: Community health worker
CI: Confidence interval
CU5: Children under 5 years
EA: Enumeration area
iCCM: Integrated community case management
mCCM: Malaria community case management
MoPH: Madagascar Ministry of Public Health
NGO: Non-governmental organization
NMCP: National Malaria Control Program
OSM: OpenStreetMap
PMI: President’s Malaria Initiative
Pro-CCM: Proactive community case management
RDT: Rapid diagnostic test
RR: Risk ratio
SSA: Sub-Saharan Africa
USAID: United States Agency for International Development
WHO: World Health Organization
