# Supplementary material for: Expanding community case management of malaria to all ages can improve universal access to malaria diagnosis and treatment: results from a cluster randomized trial in Madagascar
Source: BMC Med. 2024 Jun 10;22:231. doi: 10.1186/s12916-024-03441-9 (PMC11163690; doi:10.1186/s12916-024-03441-9)
Supplement: Supplementary file 1 — Additional file 1: Table S1. Details on primary and secondary outcomes used to evaluate the impact of age-expanded mCCM. Table S2. Health system strengthening activities and solutions implemented in both arms during mCCM expansion period. Table S3. Impact of mCCM expansion to all ages on the proportion of febrile individuals seeking care, and the proportion of RDT+ individuals receiving an ACT. Table S4. Impact of mCCM expansion to all ages on the number of febrile individuals seeking care, and the number of ACTs delivered. Table S5. Reported cost of malaria care at different levels of care for individuals who sought care. Table S6. Impact of mCCM expansion to all ages on the number of children under 5 years seeking care for diarrhea and pneumonia from CHWs. Fig S1. Comparison of observed temporal utilization patterns and multivariate model predictions. Fig S2. Comparison of observed geographic utilization patterns and multivariate model predictions. Fig S3. Changes in rates of fever care seeking by age group before and after mCCM implementation in each study arm. Fig S4. Changes in rates of fever care seeking by population distance to health centers before and after mCCM implementation in each study arm. Fig S5. Changes in rates of ACT treatments by age group before and after mCCM implementation in each study arm. Fig S6. Changes in rates of ACT treatments by population distance to health centers before and after mCCM implementation in each study arm. Fig S7. Changes in rates of ARI and diarrhea case management at community level before and after mCCM implementation in each study arm. Fig S8. RDT stocks at health facility level, before and after mCCM implementation. Fig S9. ACT stocks at health facility level, before and after mCCM implementation. Fig S10. RDT stocks at CHW level, before and after mCCM implementation. Fig S11. ACT stocks at CHW level, before and after mCCM implementation. Fig S12. Monthly stockout days at CHW level for ACTs for children <5 years, b [file 12916_2024_3441_MOESM1_ESM.docx]

Expanding community case management of malaria to all ages can improve universal access to malaria diagnosis and treatment: results from a cluster randomized trial in Madagascar

- Additional file 1 -

Andres Garchitorena^1,2*^, Aina Harimanana^2^, Judickaelle Irinantenaina^2^, Hobisoa Léa Razanadranaivo^2^, Tsinjo Fehizoro Rasoanaivo^2^, Dean Sayre^3^, Julie R. Gutman^4^, Reziky Tiandraza Mangahasimbola^2^, Masiarivony Ravaoarimanga^2^, Oméga Raobela^5^, Lala Yvette Razafimaharo^5^, Nicolas Ralemary^6^, Mahefa Andrianasolomanana^7^, Julie Pontarollo^8^, Aline Mukerabirori*^9^*, Walter Ochieng^10^, Catherine M. Dentinger^11^, Laurent Kapesa^12^, Laura C. Steinhardt^4^

*^1^ UMR MIVEGEC, IRD, CNRS, Université de Montpellier, Montpellier, France*

*^2^Unité d’épidémiologie et de recherche clinique, Institut Pasteur de Madagascar, Antananarivo, Madagascar*

*^3^U.S. President’s Malaria Initiative, Malaria Branch, Centers for Disease Control and Prevention, Atlanta, GA, USA*

*^4^ Malaria Branch, Division of Parasitic Diseases and Malaria, Centers for Disease Control and Prevention, Atlanta, GA, USA*

*^5^ Programme National de Lutte contre le Paludisme, Ministère de la Santé Publique de Madagascar, Antananarivo, Madagascar*

*^6^Direction Régionale de la Santé, Ministère de la Santé Publique, Farafangana, Madagascar*

*^7^Bureau de Santé du District, Ministère de la Santé Publique, Farafangana, Madagascar*

*^8^ONG Inter Aide, Madagascar*

*^9^ Management Sciences for Health, Antananarivo, Madagascar*

*^10^ Global Health Center, Centers for Disease Control and Prevention, Atlanta, GA, USA*

*^11^U.S. President’s Malaria Initiative, US Centers for Disease Control and Prevention, Antananarivo, Madagascar*

*^12^ U.S. President’s Malaria Initiative, USAID, Antananarivo, Madagascar*

** Corresponding author*

**Table S1.** Details on primary and secondary outcomes used to evaluate the impact of age-expanded mCCM

| **Indicator** | **Data source** | **Definition** | **Unit of analysis** | **Numerator** | **Denominator** | **Comments** |
| --- | --- | --- | --- | --- | --- | --- |
| Fever care seeking | Household survey | Proportion of individuals two months of age or older with febrile illness in the previous 2 weeks who sought care for that illness at a CHW or at a health facility | Individual | Number of individuals two months of age or older with febrile illness in the previous 2 weeks who sought care for that illness at a CHW or at a health facility by a health worker | Number of individuals two months of age or older with febrile illness in the previous 2 weeks |  |
| Fever care seeking | Health system information | Monthly per capita rate of fever cases seen by a CHW or at a health facility by a health worker | Fokontany | Monthly number of fever cases seen by a CHW or at a health facility by a health worker | Total fokontany population | Annualized rate is provided in Figure 2 for better interpretability |
| Malaria diagnosis | Household survey | Proportion of individuals two months of age or older, reporting a fever in the previous 2 weeks and who were tested with a malaria RDT by a CHW or at a health facility by a health worker | Individual | Number of individuals two months of age or older, reporting a fever in the previous 2 weeks and who were tested with a malaria RDT by a CHW or at a health facility by a health worker | Number of individuals two months of age or older with febrile illness in the previous 2 weeks |  |
| Malaria diagnosis | Health system information | Monthly per capita rate of malaria RDTs done by a CHW or at a health facility by a health worker | Fokontany | Monthly number of malaria RDTs done by a CHW or at a health facility by a health worker | Total fokontany population | Annualized rate is provided in Figure 2 for better interpretability |
| Malaria treatment | Household survey | Proportion of individuals two months of age or older with febrile illness in the previous 2 weeks who tested positive for malaria and received treatment with an appropriate antimalarial by a CHW or at a health facility by a health worker | Individual | Number of individuals two months of age or older with febrile illness in the previous 2 weeks who tested positive for malaria and received treatment with an appropriate antimalarial by a CHW or at a health facility by a health worker | Number of individuals two months of age or older with febrile illness in the previous 2 weeks who tested positive for malaria |  |
| Malaria treatment | Health system information | Monthly per capita rate of antimalarial treatments given by a CHW or at a health facility by a health worker | Fokontany | Monthly number of antimalarial treatments given by a CHW or at a health facility by a health worker | Total fokontany population | Annualized rate is provided in Figure 2 for better interpretability |
| Malaria prevalence | Household survey | Malaria parasite prevalence in children under 15 years of age as measured by malaria RDT | Individual | Number of children under 15 years of age with a positive malaria RDT | Number of children under 15 years of age who were tested with a malaria RDT during the survey |  |
| ARI care seeking | Household survey | Proportion of children under 5 years of age with an acute respiratory infection in the previous 2 weeks who sought care for that illness at a CHW or at a health facility | Individual | Number of children under 5 years of age with an acute respiratory infection in the previous 2 weeks who sought care for that illness at a CHW or at a health facility by a health worker | Number of children under 5 years of age with an acute respiratory infection in the previous 2 weeks | Acute respiratory infection is defined as cough + difficulty breathing |
| Pneumonia care seeking | Health system information | Monthly per capita rate of acute respiratory infections seen by a CHW or at a health facility by a health worker | Fokontany | Monthly number of acute respiratory infections seen by a CHW or at a health facility by a health worker | Fokontany population of children <5 years |  |
| Pneumonia treatment | Household survey | Proportion of children under 5 years of age with an acute respiratory infection in the previous 2 weeks who received antibiotic treatment by a CHW or at a health facility by a health worker | Individual | Number of children under 5 years of age with an acute respiratory infection in the previous 2 weeks who received antibiotic treatment by a CHW or at a health facility by a health worker | Number of children under 5 years of age with an acute respiratory infection in the previous 2 weeks | Acute respiratory infection is defined as cough + difficulty breathing |
| Pneumonia treatment | Health system information | Monthly per capita rate of acute respiratory infections treated with antibiotics by a CHW or at a health facility by a health worker | Fokontany | Monthly number of acute respiratory infections treated with antibiotics by a CHW or at a health facility by a health worker | Fokontany population of children <5 years |  |
| Diarrhea care seeking | Household survey | Proportion of children under 5 years of age with diarrhea in the previous 2 weeks who sought care for that illness at a CHW or at a health facility | Individual | Number of children under 5 years of age with diarrhea in the previous 2 weeks who sought care for that illness at a CHW or at a health facility | Number of children under 5 years of age with diarrhea in the previous 2 weeks |  |
| Diarrhea care seeking | Health system information | Monthly per capita rate of diarrhea cases seen by a CHW or at a health facility by a health worker | Fokontany | Monthly number of diarrhea cases seen by a CHW or at a health facility by a health worker | Fokontany population of children <5 years |  |
| Diarrhea treatment | Household survey | Proportion of children under 5 years of age with diarrhea in the previous 2 weeks who received oral rehydration salt treatment at a CHW or at a health facility | Individual | Number of children under 5 years of age with diarrhea in the previous 2 weeks who received oral rehydration salt treatment by a CHW or at a health facility by a health worker | Number of children under 5 years of age with diarrhea in the previous 2 weeks | Insufficient numbers of reported diarrhea prevented estimation of this indicator |
| Diarrhea treatment | Health system information | Monthly per capita rate of diarrhea cases treated with oral rehydration salts by a CHW or at a health facility by a health worker | Fokontany | Monthly number of diarrhea cases treated with oral rehydration salts by a CHW or at a health facility by a health worker | Fokontany population of children <5 years |  |

**Table S2.** Health system strengthening activities and solutions implemented in both arms during mCCM expansion period

| **Type of challenge** | **Problem observed** | **Health system strengthening activities and solutions** |
| --- | --- | --- |
| **Supply chain management** |  |  |
| Stock quantification | Uncertainty around stocks needed for mCCM expansion given the lack of baseline  Use of supplies substantially larger than initially expected | Collaboration with National Committee for procurement of supplies, stock and management (“GAS” committee) for initial quantification and training  Emergency orders to limit stock-outs |
| Storage of supplies | Insufficient space at district pharmacy for additional supply needs | Rental of additional storage space in Farafangana |
| Supply distribution from central to district level | Delays in distribution of routine and emergency orders | Coordination with GAS committee; transport of emergency orders by study teams |
| Supply distribution from district to health center level | Pushback from heads of health centers to transport additional CHW supplies to their health centers given additional cost and lack of means necessary | Transport by study teams whenever possible, both during monthly supervision and for emergency orders  Accompaniment to obtain last mile transport incentives from existing national programs |
| Supply distribution from health center to CHW level | Pushback from heads of health centers to distribute supplies to CHWs even if available at health center | Accompaniment of CHW supply order by study team during monthly review  Separate supply orders (earmarked) for health centers and CHWs |
| Complex and time consuming reporting process | Multiplicity of paper forms for CHWs to fill (stock management, register for children under 5 years and individuals over 5 years, monthly activity report, supply orders) | CHW training, monthly supervision and on-demand coaching sessions by study team |
| **Malaria care free of charge** |  |  |
| Payment for malaria supplies | Informal payments requested at CHWs or health centers for malaria care | Community sensitisation around malaria care being free of charge (radio, community meetings) |
| CHW incentives | Ban to sell malaria supplies and increase in workload | Payment of an incentive every six months to every CHW, of 10,000 ariary/CHW/month |
| Corruption issues | Sale of MNCP's malaria supplies in certain district markets | Investigation by district officials and local police |
| **Coordination with Ministry of Health** |  |  |
| Coordination between national, district & regional level | Lack of initial MoPH involvement for problem resolution  Difficulty in communication across levels of health system | Monthly coordination meetings with MoPH representatives at central (MNCP coordinator), regional (director), and district (medical inspector) level |

**Table S3.** Impact of mCCM expansion to all ages on i) the proportion of febrile individuals seeking care, and ii) the proportion of RDT+ individuals receiving an ACT (logistic regression, difference-in-differences analyses using survey data). Similar results for the number of RDTs done are presented in the main text.

| **Age group** | **Level of care** | **Intercept** | **Change over time (ref. baseline)** | **Arm differences (ref. control arm)** | **Difference-in-differences  (Period X Arm)** | **Observations** |
| --- | --- | --- | --- | --- | --- | --- |
|  |  |  | **OR (95% CI)** | **OR (95% CI)** | **OR (95% CI)** |  |
| **Fever care seeking** |  |  |  |  |  |  |
| All ages | Both levels of care | 0.27 (0.19-0.38)*** | 9.36 (4.61-19.01)*** | 0.92 (0.42-2.04) | 0.58 (0.2-1.64) | 717 |
|  | Health center | 0.17 (0.1-0.28)*** | 3.36 (1.14-9.88)* | 1 (0.3-3.38) | 0.42 (0.1-1.8) | 717 |
|  | CHW | 0.12 (0.07-0.21)*** | 4.66 (1.76-12.38)** | 0.75 (0.3-1.9) | 1.62 (0.49-5.3) | 717 |
| Children 0-5 years | Both levels of care | 0.29 (0.17-0.45)* | 30.24 (9-101.56)*** | 0.97 (0.42-2.24) | 0.2 (0.04-0.89)* | 261 |
|  | Health center | 0.19 (0.09-0.34)*** | 2.96 (0.61-14.33) | 0.94 (0.3-2.91) | 0.5 (0.09-2.71) | 261 |
|  | CHW | 0.12 (0.06-0.23)*** | 8.27 (2.68-25.57)*** | 0.91 (0.27-3.02) | 0.99 (0.21-4.58) | 261 |
| Children 6-13 years | Both levels of care | 0.28 (0.16-0.45)* | 16.86 (2.99-95.11)** | 0.74 (0.25-2.25) | 0.47 (0.06-3.53) | 229 |
|  | Health center | 0.16 (0.09-0.28)*** | 7.28 (2.01-26.4)** | 0.77 (0.16-3.65) | 0.21 (0.03-1.6) | 229 |
|  | CHW | 0.16 (0.07-0.34)** | 2.29 (0.7-7.48) | 0.61 (0.17-2.24) | 3.86 (0.78-19.23) | 229 |
| Invididuals 14+ years | Both levels of care | 0.24 (0.16-0.35)*** | 2.28 (0.67-7.78) | 1.17 (0.42-3.26) | 1.49 (0.27-8.13) | 226 |
|  | Health center | 0.16 (0.09-0.28)*** | 1.31 (0.47-3.69) | 1.46 (0.39-5.47) | 0.84 (0.16-4.39) | 226 |
|  | CHW | 0.09 (0.04-0.22)*** | 4.04 (0.72-22.76) | 0.76 (0.18-3.13) | 1.55 (0.18-13.08) | 226 |
| **ACT among RDT+** |  |  |  |  |  |  |
| All ages | Both levels of care | 0.47 (0.23-0.73) | 7.65 (1.73-33.91)** | 1.49 (0.38-5.84) | 1.86 (0.29-12.01) | 234 |
|  | Health center | 0.33 (0.15-0.58) | 1.89 (0.4-8.98) | 1.49 (0.31-7.22) | 0.33 (0.05-2.08) | 234 |
|  | CHW | 0.16 (0.05-0.4)* | 4.36 (1-19.06). | 1.22 (0.23-6.31) | 2.01 (0.32-12.61) | 234 |
| Children 0-5 years | Both levels of care | 0.46 (0.21-0.73) | 15.99 (2.38-107.42)** | 1.13 (0.27-4.69) | 1.11 (0.08-14.71) | 90 |
|  | Health center | 0.31 (0.13-0.59) | 0.99 (0.14-7.22) | 0.94 (0.16-5.6) | 1.03 (0.1-10.19) | 90 |
|  | CHW | 0.14 (0.05-0.36)** | 9.71 (1.91-49.28)** | 1.38 (0.21-8.85) | 1.05 (0.09-12.45) | 90 |
| Children 6-13 years | Both levels of care | 0.42 (0.18-0.7) | 12.91 (1.95-85.44)* | 2.58 (0.37-17.96) |  | 90 |
|  | Health center | 0.28 (0.12-0.54). | 5.46 (1.02-29.31). | 2.55 (0.33-19.6) | 0.06 (0-0.84)* | 90 |
|  | CHW | 0.14 (0.03-0.44)* | 2.28 (0.35-14.78) | 1.69 (0.2-14.35) | 4.73 (0.34-66) | 90 |
| Invididuals 14+ years | Both levels of care | 0.68 (0.18-0.96) | 0.82 (0.06-10.81) | 0.59 (0.05-6.99) | 8.54 (0.49-150.28) | 53 |
|  | Health center | 0.55 (0.17-0.88) | 0.56 (0.07-4.66) | 0.8 (0.11-5.97) | 1.21 (0.12-12.28) | 53 |
|  | CHW | 0.27 (0.03-0.79) | 2.59 (0.2-33.13) | 0.44 (0.02-8.1) | 2.73 (0.12-64.71) | 53 |

*p<0.05; **p<0.01; ***p<0.001

**Table S4.** Impact of mCCM expansion to all ages on i) the number of febrile individuals seeking care, and ii) the number of ACTs delivered (negative binomial regression, interrupted time-series analyses^1^ using health system data). Similar results for the number of RDTs done are presented in the main text.

| **Age group** | **Level of care** | **Arm differences  (ref. control)** | **Change over time (ref. before)** | **Impact of mCCM (level of change)** | **Impact of mCCM (slope of change)** | **Impact of mCCM over distance to HF (km)** |
| --- | --- | --- | --- | --- | --- | --- |
|  |  | **RR (95% CI)** | **RR (95% CI)** | **RR (95% CI)** | **RR (95% CI)** | **RR (95% CI)** |
| **Rates of fever cases seen per month** | | | | | | |
| All ages | Both levels of care | 0.96 (0.83-1.1) | 1.94 (1.85-2.04)*** | 0.97 (0.9-1.05) | 0.99 (0.92-1.07) | 1.07 (1.06-1.08)*** |
| All ages | Health center level | 0.89 (0.7-1.13) | 1.18 (1.1-1.27)*** | 1.02 (0.91-1.15) | 0.75 (0.67-0.83)*** | 1.02 (1.01-1.04)** |
| All ages | CHW level | 1.07 (0.95-1.2) | 2.77 (2.62-2.93)*** | 1.37 (1.25-1.49)*** | 1.03 (0.95-1.11) | 1.01 (1-1.02)* |
| Children 0-5 years | Both levels of care | 0.98 (0.85-1.12) | 2.05 (1.95-2.15)*** | 0.75 (0.69-0.81)*** | 0.98 (0.91-1.05) | 1.03 (1.02-1.04)*** |
| Children 6-13 years | Both levels of care | 1.1 (0.88-1.39) | 1.27 (1.17-1.38)*** | 1.91 (1.67-2.18)*** | 1.09 (0.98-1.22) | 1.22 (1.2-1.24)*** |
| Invididuals 14+ years | Both levels of care | 1.04 (0.84-1.29) | 1.27 (1.18-1.37)*** | 1.71 (1.51-1.93)*** | 1.02 (0.92-1.13) | 1.2 (1.18-1.22)*** |
| **Rates of ACTs given per month** | | | | | | |
| All ages | Both levels of care | 0.99 (0.84-1.17) | 2.52 (2.36-2.69)*** | 1.03 (0.92-1.14) | 0.88 (0.81-0.97)* | 1.09 (1.07-1.1)*** |
| All ages | Health center level | 1.06 (0.81-1.38) | 0.95 (0.88-1.02) | 1.24 (1.1-1.4)*** | 0.47 (0.42-0.52)*** | 1.02 (1-1.04)* |
| All ages | CHW level | 1.02 (0.88-1.19) | 9.23 (8.24-10.33)*** | 1.23 (1.03-1.47)* | 1.63 (1.39-1.92)*** | 1.01 (0.99-1.04) |
| Children 0-5 years | Both levels of care | 0.98 (0.82-1.16) | 3.33 (3.1-3.59)*** | 0.78 (0.7-0.88)*** | 0.95 (0.85-1.05) | 1.04 (1.03-1.06)*** |
| Children 6-13 years | Both levels of care | 1.31 (1.02-1.68)* | 1.07 (0.98-1.16) | 1.77 (1.55-2.02)*** | 0.66 (0.59-0.74)*** | 1.21 (1.18-1.23)*** |
| Invididuals 14+ years | Both levels of care | 1.26 (1-1.58)* | 1.25 (1.15-1.36)*** | 1.6 (1.41-1.82)*** | 0.71 (0.64-0.79)*** | 1.19 (1.17-1.22)*** |

^1^ Analyses were controlled for linear time trends, seasonality, lagged utilization (t-1 month) and a non-linear smooth for distance from fokontany to nearest health center.

*p<0.05; **p<0.01; ***p<0.001


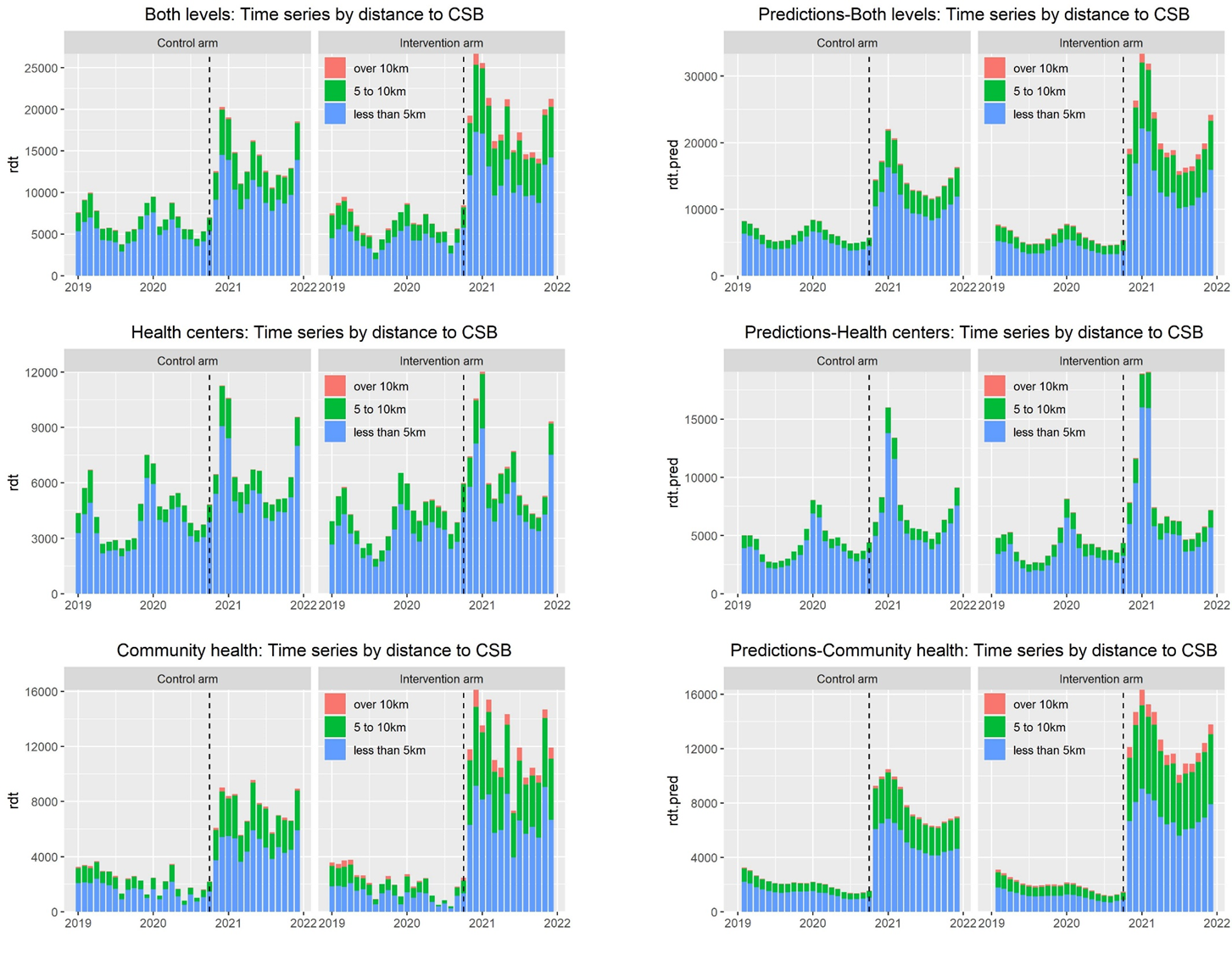


**Figure S1. Comparison of observed temporal utilization patterns and multivariate model predictions.** Results show that our models predicted well (right) the time series of RDTs done per month observed (left) at different distances, both in the control and intervention arms and for each level of care, during the study period.


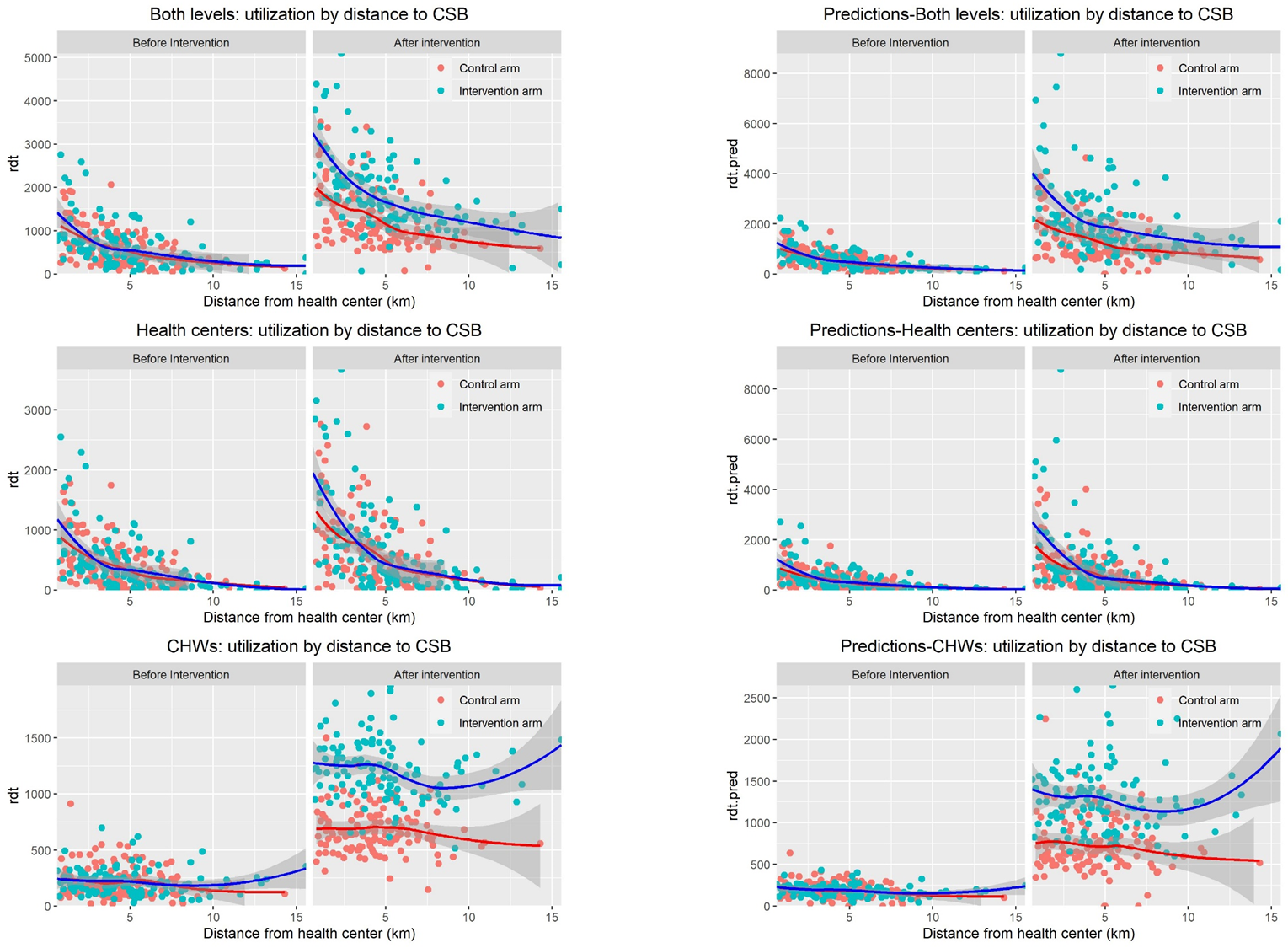


**Figure S2. Comparison of observed geographic utilization patterns and multivariate model predictions.** Results show that our models predicted well (right) the geographic patterns of RDTs done observed (left) at different distances, both in the control and intervention arms and for each level of care, during the study period.

**
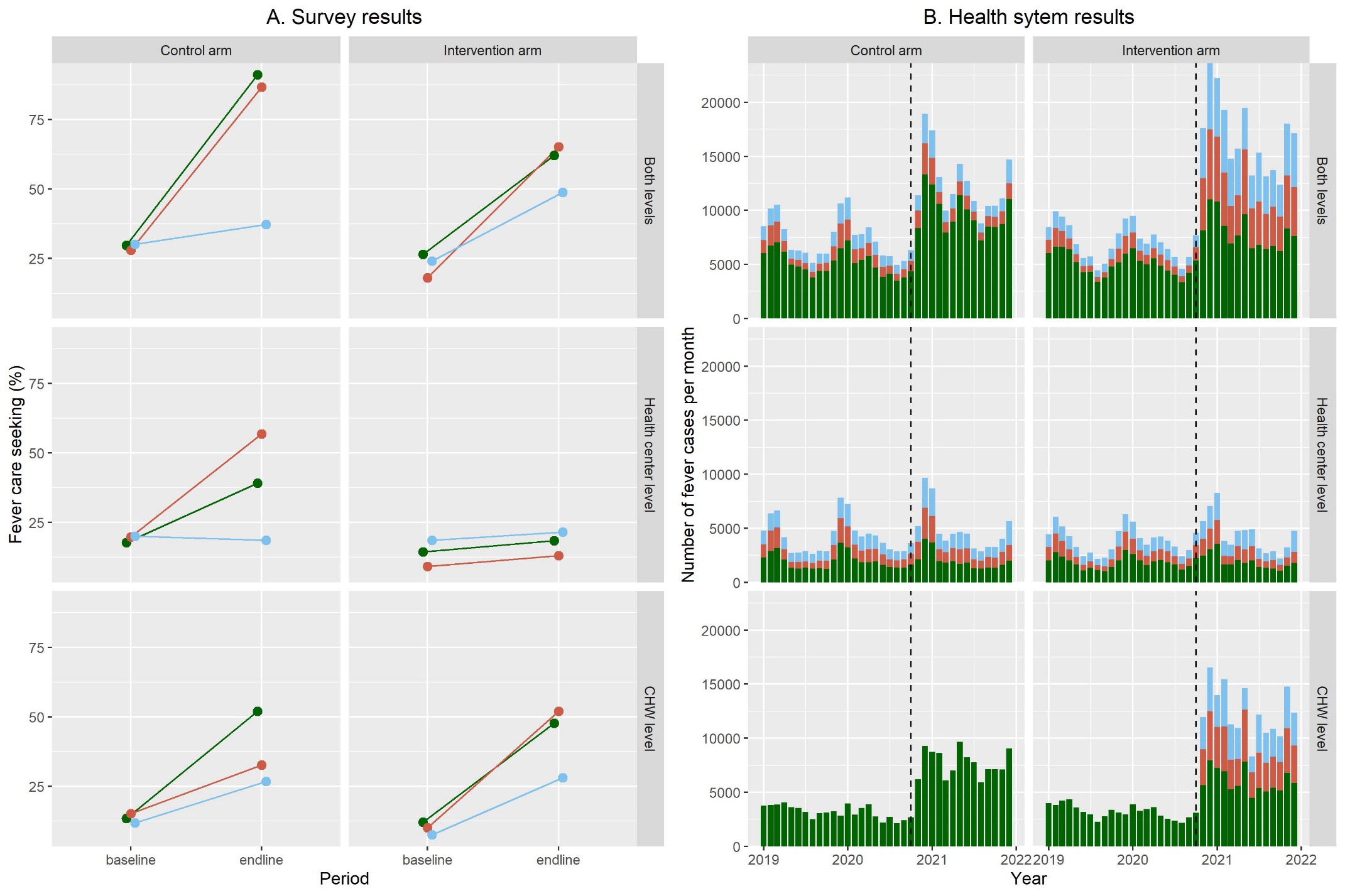
**

**Figure S3. Changes in rates of fever care seeking by age group before and after mCCM implementation in each study arm.** A) Results from household surveys, comprising individuals who declared being ill in the previous two weeks. B) Results from health system information, comprising monthly primary care consultations at health centers and CHWs.

**
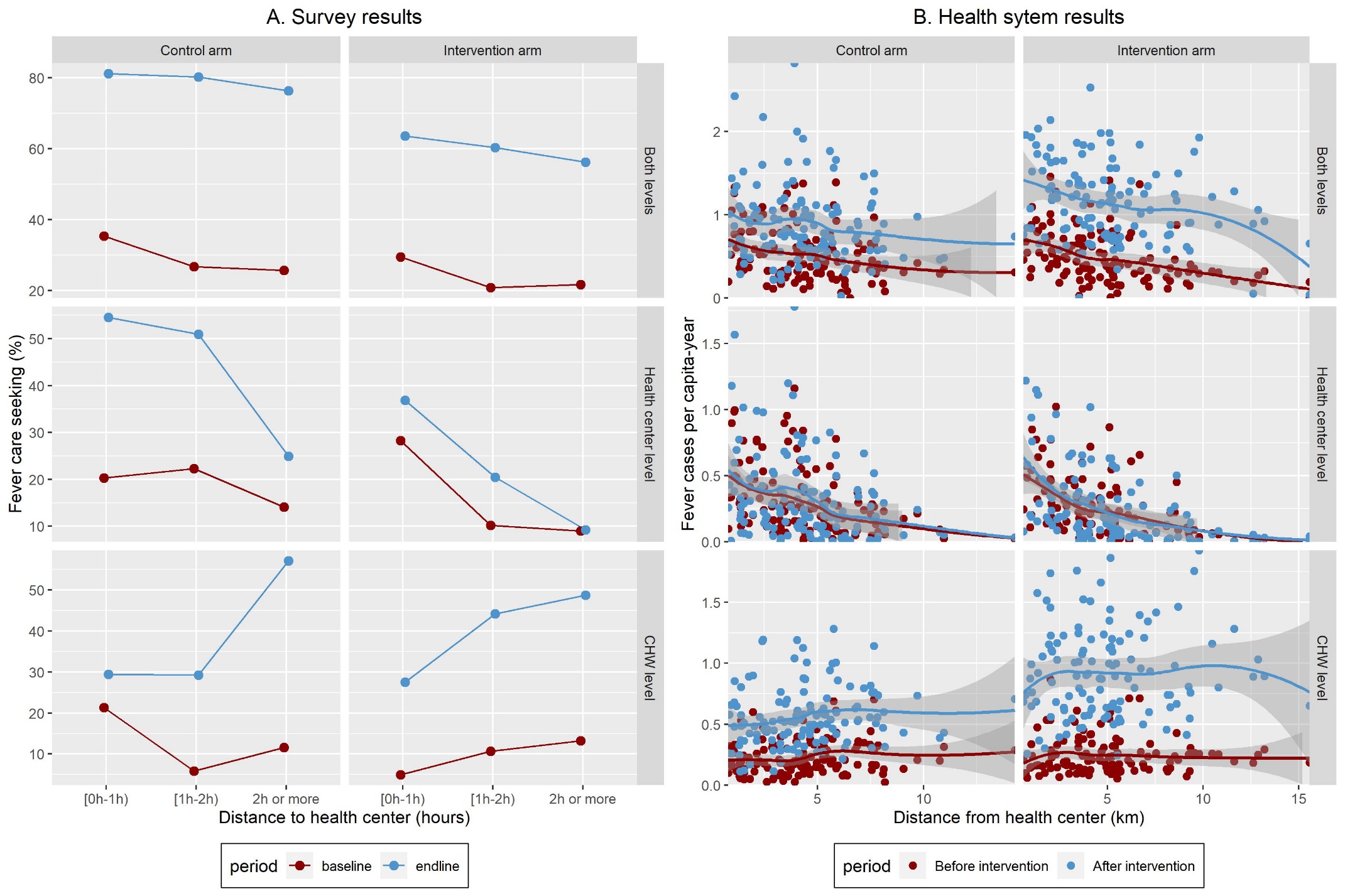
**

**Figure S4. Changes in rates of fever care seeking by population distance to health centers before and after mCCM implementation in each study arm.** A) Results from household surveys, comprising individuals who declared being ill in the previous two weeks and reported travel time to the nearest health center. B) Results from health system information, comprising monthly primary care consultations at health centers and CHWs and estimated distance to nearest health center via OSRM. Each dot represents the average of one Fokontany, with solid lines representing the fitted smooth from a general additive model and its 95% confidence intervals (grey area).

**
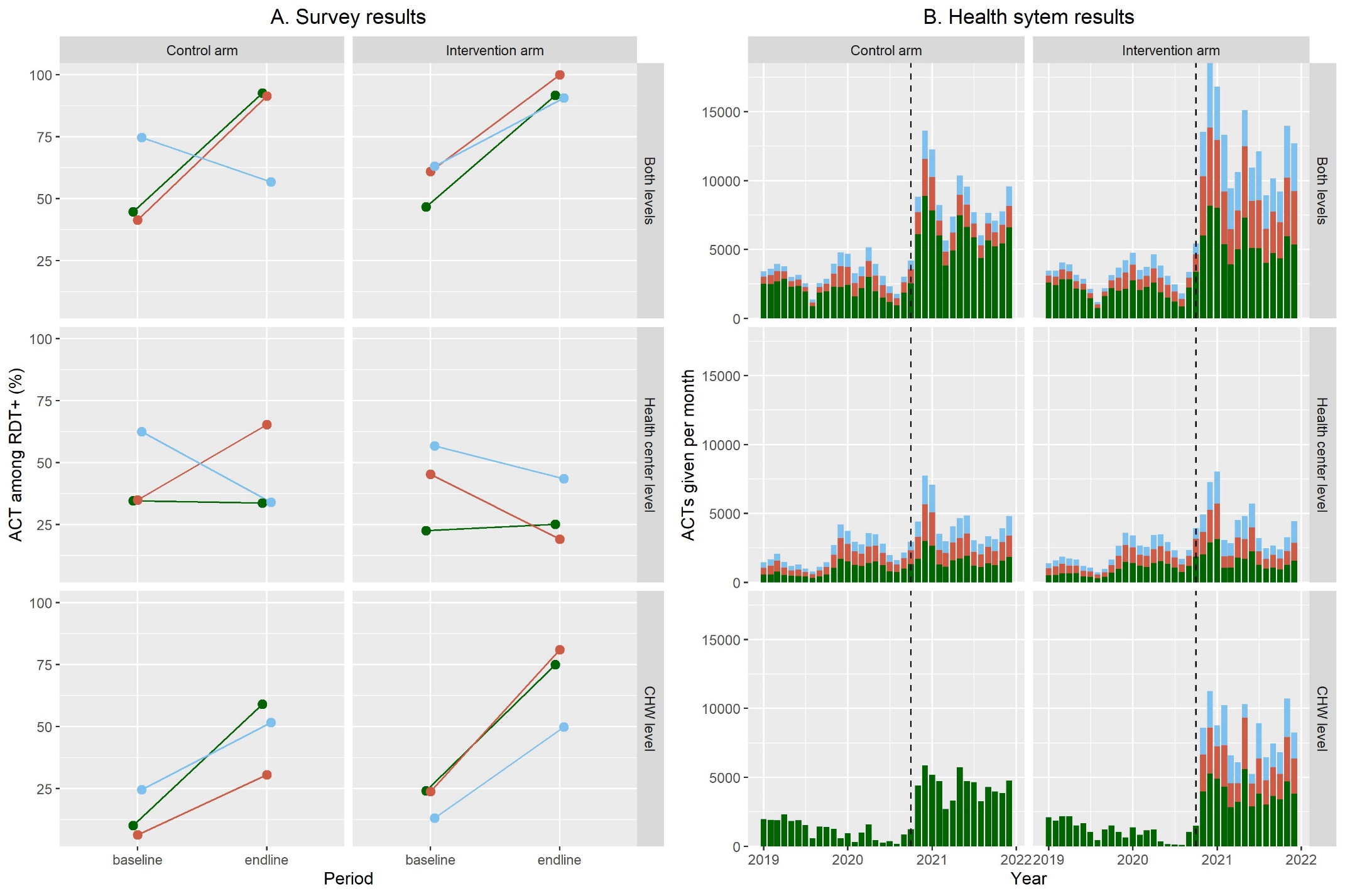
**

**Figure S5. Changes in rates of ACT treatments by age group before and after mCCM implementation in each study arm.** A) Results from household surveys, comprising individuals who declared having a positive RDT in the previous two weeks. B) Results from health system information, comprising monthly primary care consultations at health centers and CHWs.

**
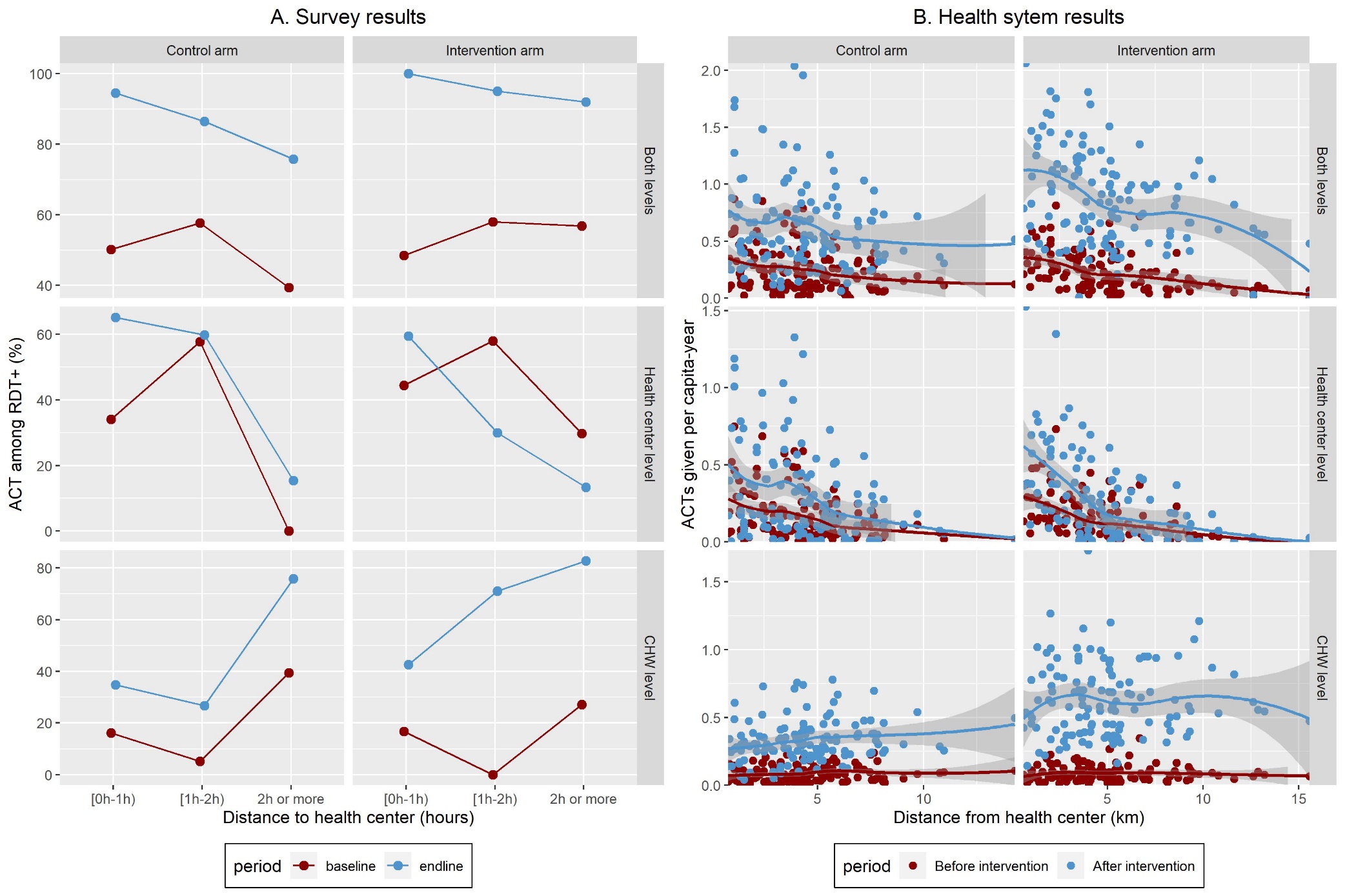
**

**Figure S6. Changes in rates of ACT treatments by population distance to health centers before and after mCCM implementation in each study arm.** A) Results from household surveys, comprising individuals who declared having a positive RDT in the previous two weeks and reported travel time to the nearest health center. B) Results from health system information, comprising monthly primary care consultations at health centers and CHWs and estimated distance to nearest health center via OSRM. Each dot represents the average of one Fokontany, with solid lines representing the fitted smooth from a general additive model and its 95% confidence intervals (grey area).

**Table S5.** Reported cost of malaria care at different levels of care for individuals who sought care (analyses of survey data)

| **Variable** | **Baseline (N=110)** | **Endline (N=242)** |
| --- | --- | --- |
|  | **Average (95% CI)** | **Average (95% CI)** |
| **Both levels of care** |  |  |
| Payment among those who sought care, % | 94.1 (88.9-97) | 72.1 (63.5-79) |
| Average cost, ariary | 3346 (2514.37-4177.67) | 3981.6 (2719.08-5244.15) |
| Payment for consultation, % | 17.55 (8.54-33) | 16.9 (10.6-26) |
| Payment for mRDT, % | 59.9 (36.6-80) | 36.2 (25.2-49) |
| Payment for ASAQ, % | 94.5 (78.1-99) | 98.4 (93.4-100) |
| **Health center level** |  |  |
| Payment among those who sought care, % | 96.3 (86.7-99) | 90.2 (76.7-96) |
| Average cost, ariary | 3585.1 (2655.96-4514.17) | 3920.5 (3206.63-4634.45) |
| Payment for consultation, % | 21.9 (7.08-51) | 19.2 (7.11-42) |
| Payment for mRDT, % | 38.4 (14.6-70) | 26.7 (11.2-51) |
| Payment for ASAQ, % | 97 (81.1-100) | 100 |
| **CHW level** |  |  |
| Payment among those who sought care, % | 93.3 (79.2-98) | 60.2 (50.6-69) |
| Average cost, ariary | 2514 (1778.17-3249.92) | 2526.3 (1979.23-3073.46) |
| Payment for consultation, % | 9.71 (2.94-28) | 21.4 (10.5-39) |
| Payment for mRDT, % | 83.7 (61.9-94) | 40.4 (25.9-57) |
| Payment for ASAQ, % | 86.6 (46.7-98) | 96.8 (86.8-99) |


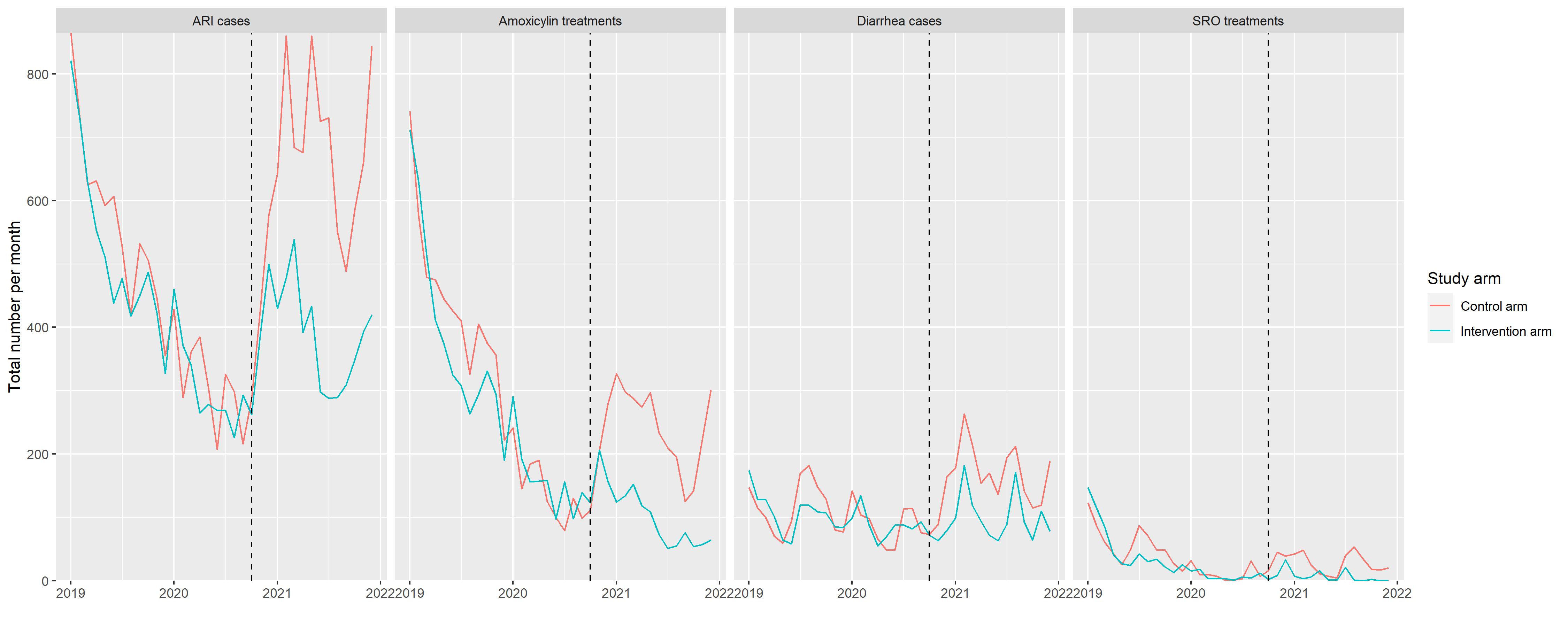


**Figure S7. Changes in rates of ARI and diarrhea case management at community level before and after mCCM implementation in each study arm.** Results from health system information, comprising total monthly cases seen and treatments given for ARI and diarrhea by CHWs for children under 5 years.

**Table S6.** Impact of mCCM expansion to all ages on the number of children under 5 years seeking care for diarrhea and pneumonia from CHWs (negative binomial regression, interrupted time-series analyses^1^ using health system data)

| **Variable** | **Arm differences  (ref. control)** | **Change over time (ref. before)** | **Impact of mCCM (level of change)** |
| --- | --- | --- | --- |
|  | **RR (95% CI)** | **RR (95% CI)** | **RR (95% CI)** |
| Rates of ARI cases seen | 0.72 (0.55-0.95)* | 2.51 (2.24-2.81)*** | 0.66 (0.59-0.73)*** |
| Rates of Amoxicylin treatments given | 0.75 (0.55-1.02) | 3.14 (2.62-3.76)*** | 0.37 (0.31-0.44)*** |
| Rates of diarrhea cases seen | 0.74 (0.52-1.04) | 1.91 (1.62-2.26)*** | 0.74 (0.63-0.87)*** |
| Rates of SRO treatments given | 0.71 (0.46-1.11) | 7.18 (4.81-10.73)*** | 0.40 (0.26-0.61)*** |

^1^ Analyses were controlled for linear time trends, lagged utilization (t-1 month) and a non-linear smooth for distance from fokontany to nearest health center.

*p<0.05; **p<0.01; ***p<0.001


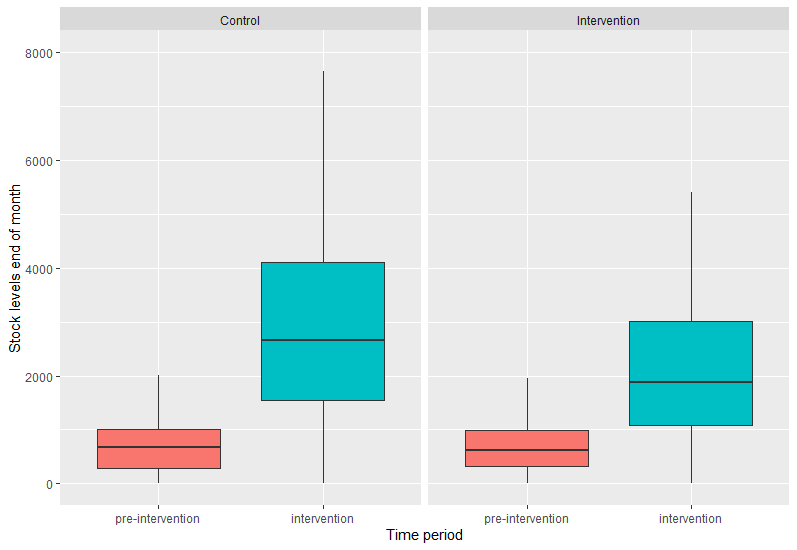


**Figure S8: RDT stocks at health facility level, before and after mCCM implementation.** Data include monthly stock levels (at the end of the month) at each of the 30 study facilities pre-intervention (January 2019 – October 2020) and during mCCM intervention (November 2020 – December 2021). Control facilities are on the left and intervention facilities are on the right. The intervention period during mCCM is represented by the box plots in blue. Outliers have been removed.


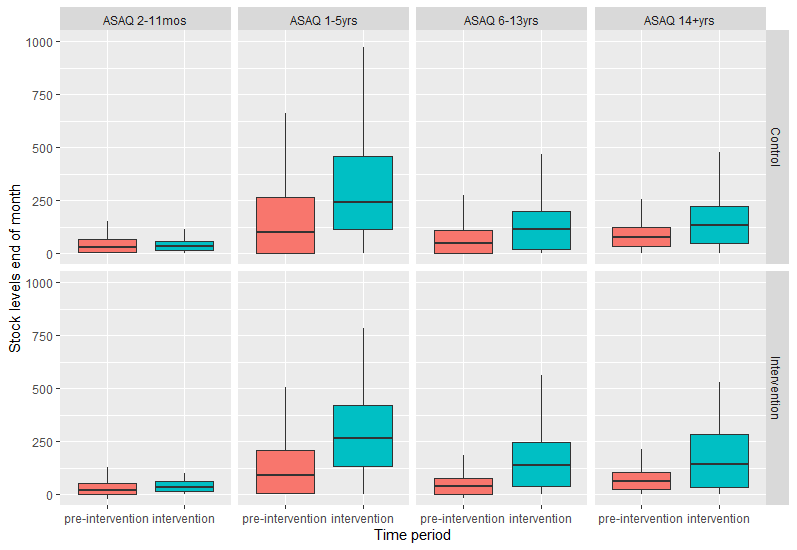


**Figure S9: ACT stocks at health facility level, before and after mCCM implementation.** Data include monthly stock levels (at the end of the month) at each of the 30 study facilities pre-intervention (January 2019 – October 2020) and during mCCM intervention (November 2020 – December 2021). Control facilities are on the left and intervention facilities are on the right. The intervention period during mCCM is represented by the box plots in blue. Outliers have been removed.


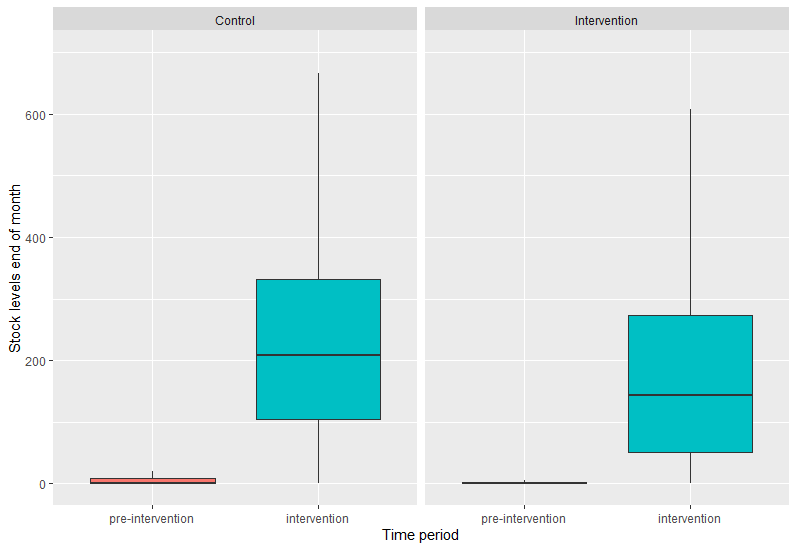


**Figure S10: RDT stocks at CHW level, before and after mCCM implementation.** Data include monthly stock levels (at the end of the month) at each of the ~500 CHWs pre-intervention (January 2020 – October 2020) and during mCCM intervention (November 2020 – December 2021). Control CHWs are on the left and intervention CHWs are on the right. The intervention period during mCCM is represented by the box plots in blue. Outliers have been removed.


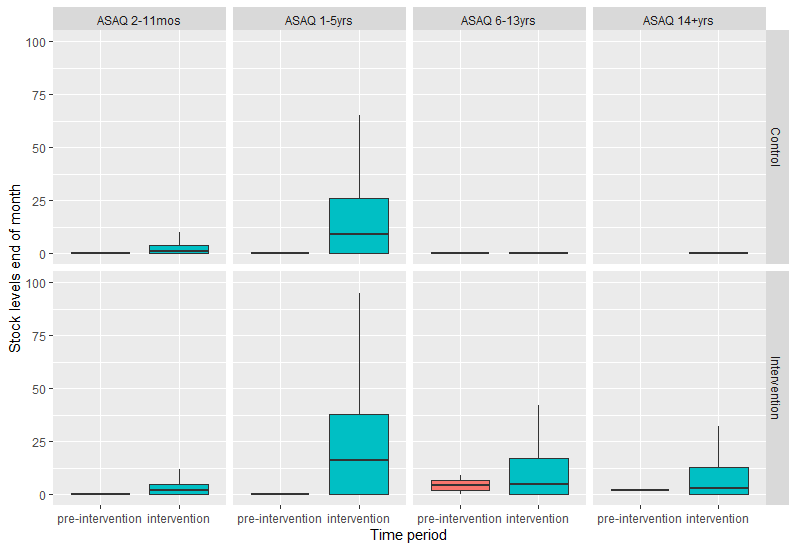


**Figure S11: ACT stocks at CHW level, before and after mCCM implementation.** Data include monthly stock levels (at the end of the month) at each of the ~500 CHWs pre-intervention (January 2020 – October 2020) and during mCCM intervention (November 2020 – December 2021). Control CHWs are on the left and intervention CHWs are on the right. The intervention period during mCCM is represented by the box plots in blue. Outliers have been removed.


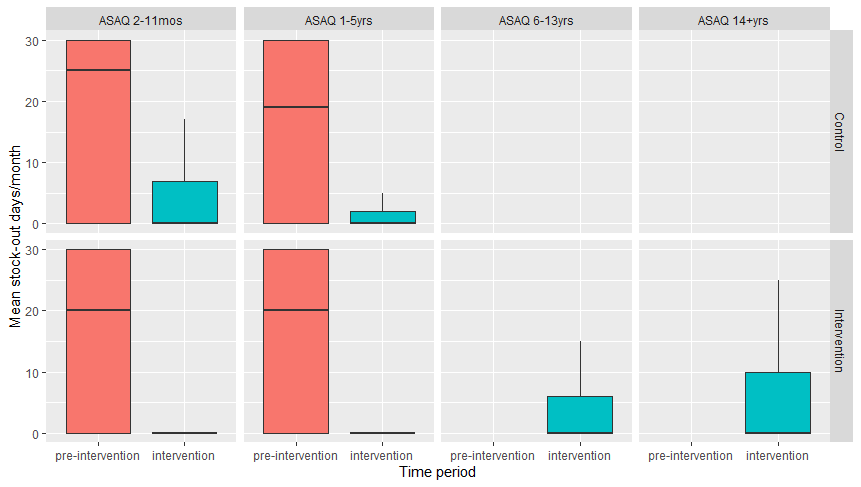


**Figure S12: Monthly stockout days at CHW level for ACTs for children <5 years, before and after mCCM implementation.** Data include monthly stockout days at each of the ~500 CHWs pre-intervention (January 2020 – October 2020) and during mCCM intervention (November 2020 – December 2021). Control CHWs are on the left and intervention CHWs are on the right. The intervention period during mCCM is represented by the box plots in blue. Outliers have been removed.


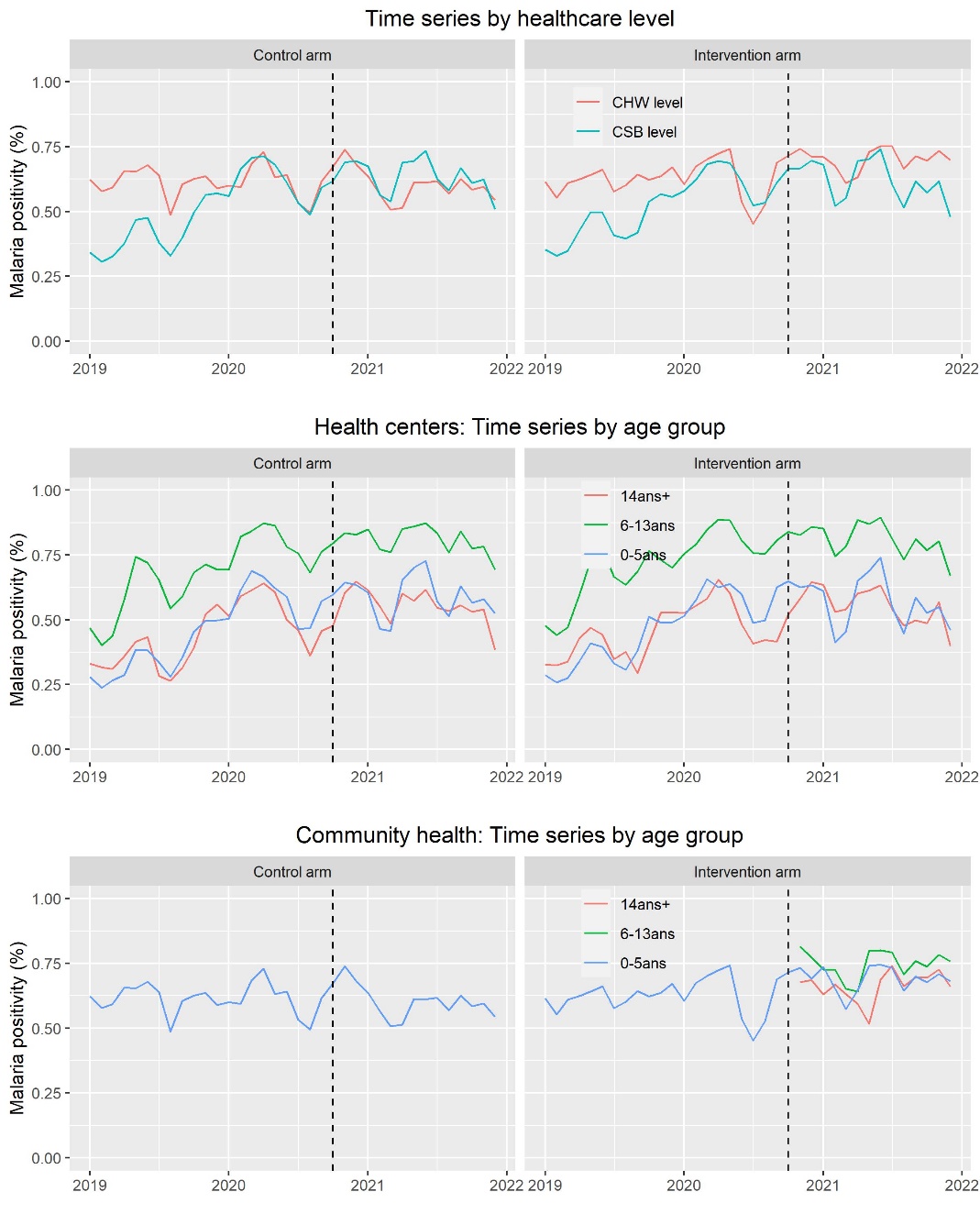


**Figure S13. Evolution of RDT positivity over time in Farafangana District, 2019-2021.** Results from health system information, comprising monthly primary care consultations at health centers and CHWs.
