# Supplementary material for: Expanding community case management of malaria to all ages can improve universal access to malaria diagnosis and treatment: results from a cluster randomized trial in Madagascar
Source: BMC Med. 2024 Jun 10;22:231. doi: 10.1186/s12916-024-03441-9 (PMC11163690; doi:10.1186/s12916-024-03441-9)
Supplement: Supplementary file 2 — Additional file 2: Supplementary analyses on intervention costing. Table S7. Data Sources for the different categories included in the costing analysis. Table S8. Average costs of diagnosis and treatment of suspected cases of uncomplicated malaria at the community level. [file 12916_2024_3441_MOESM2_ESM.docx]

Expanding community case management of malaria to all ages can improve universal access to malaria diagnosis and treatment: results from a cluster randomized trial in Madagascar

- Additional file 2 -

Andres Garchitorena^1,2*^, Aina Harimanana^2^, Judickaelle Irinantenaina^2^, Hobisoa Léa Razanadranaivo^2^, Tsinjo Fehizoro Rasoanaivo^2^, Dean Sayre^3^, Julie R. Gutman^4^, Reziky Tiandraza Mangahasimbola^2^, Masiarivony Ravaoarimanga^2^, Oméga Raobela^5^, Lala Yvette Razafimaharo^5^, Nicolas Ralemary^6^, Mahefa Andrianasolomanana^7^, Julie Pontarollo^8^, Aline Mukerabirori*^9^*, Walter Ochieng^10^, Catherine M. Dentinger^11^, Laurent Kapesa^12^, Laura C. Steinhardt^4^

*^1^ UMR MIVEGEC, IRD, CNRS, Université de Montpellier, Montpellier, France*

*^2^Unité d’épidémiologie et de recherche clinique, Institut Pasteur de Madagascar, Antananarivo, Madagascar*

*^3^U.S. President’s Malaria Initiative, Malaria Branch, Centers for Disease Control and Prevention, Atlanta, GA, USA*

*^4^ Malaria Branch, Division of Parasitic Diseases and Malaria, Centers for Disease Control and Prevention, Atlanta, GA, USA*

*^5^ Programme National de Lutte contre le Paludisme, Ministère de la Santé Publique de Madagascar, Antananarivo, Madagascar*

*^6^Direction Régionale de la Santé, Ministère de la Santé Publique, Farafangana, Madagascar*

*^7^Bureau de Santé du District, Ministère de la Santé Publique, Farafangana, Madagascar*

*^8^ONG Inter Aide, Madagascar*

*^9^ Management Sciences for Health, Antananarivo, Madagascar*

*^10^ Global Health Center, Centers for Disease Control and Prevention, Atlanta, GA, USA*

*^11^U.S. President’s Malaria Initiative, US Centers for Disease Control and Prevention, Antananarivo, Madagascar*

*^12^ U.S. President’s Malaria Initiative, USAID, Antananarivo, Madagascar*

** Corresponding author*

Supplementary analyses: intervention costing

While a detailed cost-effectiveness analysis of the age-expanded mCCM intervention was beyond the scope of this article, we used an ingredients-based costing approach to evaluate the budgetary impact of implementing the age-expanded mCCM program in Farafangana district from a health system perspective. Primary data were gathered through interviews with government officials, including medical supply chain and national malaria program managers, and medical supply data from USAID implementing partners as well as national and international pharmaceutical suppliers to Madagascar (Table S7). Secondary data were extracted from the mCCM program budgetary reports and invoices, published literature, and the World Health Organization Choosing Interventions that are Cost-Effective project (WHO-CHOICE). The numbers of patients seen by CHWs were extracted from monthly reports to the district as described in the main text.

**Table S7: Data Sources for the different categories included in the costing analysis**

| **Category** | **Data source** |
| --- | --- |
| Personnel | Madagascar Ministry of Public Health, national level  IMPACT program (PMI-funded supply chain strengthening program operating in Madagascar) |
| Consumables | IMPACT program (PMI-funded supply chain strengthening program operating in Madagascar)  ARTEMIS (USAID ordering system)  Salama (National pharmaceutical supplier in Madagascar)  PSM (USAID implementing partner for procurement of medical/prevention supplies for malaria and other diseases) |
| Training | IMPACT program (PMI-funded supply chain strengthening program operating in Madagascar)  Institut Pasteur Madagascar (Lead institution responsible for the age-expanded mCCM trial)  Madagascar Ministry of Public Health, district health office |
| Monitoring and Evaluation | IMPACT program (PMI-funded supply chain strengthening program operating in Madagascar)  Institut Pasteur Madagascar (Lead institution responsible for the age-expanded mCCM trial) |
| Planning | IMPACT program (PMI-funded supply chain strengthening program operating in Madagascar) |
| Transportation | Institut Pasteur Madagascar (Lead institution responsible for the age-expanded mCCM trial) |
| Community sensitization | Institut Pasteur Madagascar (Lead institution responsible for the age-expanded mCCM trial) |
| Support supervision | Institut Pasteur Madagascar (Lead institution responsible for the age-expanded mCCM trial) |

The costs covered fixed and variable expenditures associated with starting and scaling up the age-expanded mCCM program including personnel training, planning, community sensitization, consumables, supervision, and equipment purchases. Research-related costs were excluded. Costs covered a one-year operational horizon with a base year of 2021. Capital costs, including motor vehicle and motorcycle procurement costs were annualized using straight line depreciation using a discount rate of 3%. We assumed a wastage rate of 5% for consumables.

Sensitivity analyses were conducted to assess the impact of variations in key cost drivers on expenditure estimates. Detailed economic analyses including costing of CHW time, household costs and productivity impacts will be included in a forthcoming paper.

We used an exchange rate of 3,924.20 Ariary per USD for costs that were measured in local currency. This corresponds to the exchange rate as of January 2022^[[1]](#footnote-1)^ (end of period considered in this analysis), which had been stable for 2021 (exchange rate in January 2021 was 3,905.54 Ariary per USD). We used a time horizon of one year and consequently did not discount these costs. We also did not annuitize the costs of equipment like thermometers because we assumed their mean lifespan was under one year.

Results

We estimated the total cost of running the expanded program at $794,270 per year in the district’s study areas (Table S8). This translates to approximately $2.55 per case of suspected uncomplicated malaria managed in the community.

**Table S8: Average costs of diagnosis and treatment of suspected cases of uncomplicated malaria at the community level**

| **Category** | **Per Patient** | **Total** | **Percentage** |
| --- | --- | --- | --- |
| Staff | $0.01 | $1,780.99 | 0.2% |
| RDT and related costs (gloves, lancets, etc.) | $1.98 | $616,694.04 | 77.6% |
| Drugs (antimalarials + analgesics) | $0.42 | $130,710.51 | 16.5% |
| Equipment (thermometers, etc.) | $0.06 | $18,710.34 | 2.4% |
| Capital (vehicle, motorcycles) | $0.03 | $10,679.61 | 1.3% |
| Planning (National and District) | $0.01 | $4,217.89 | 0.5% |
| Supervision (National and District) | $0.02 | $6,975.55 | 0.9% |
| Training including refreshers | $0.02 | $5,099.89 | 0.6% |
| Community sensitization | $0.00 | $1,182.91 | 0.1% |
| **Total** | **$2.55** | **$794,270.74** | **100%** |

- RDT costs include gloves, lancets, alcohol handrub, waste disposal, and alcohol swabs. We also include the costs of transport and storage as part of the direct consumable costs. We use an RDT price of $1.05 per kit. And then adjust this for wastage at 5% before adding the costs of other consumables.
- Drugs and consumables account for 94% of the costs followed by capital costs and program management costs (staff plus supervision).
- Capital costs include a new vehicle and two motorcycles that were purchased for the program. We assume that the vehicle and motorcycles will be used exclusively to support community case management, which was the case of the mCCM trial but might not be part of routine scale-up.
- The costs assume that the CHWs were working exclusively in malaria case management. If the costs are adjusted to reflect other responsibilities, i.e., that costs of capital, planning, training, and supervision are adjusted to include care for other conditions a CHW might manage such as respiratory illnesses among young children or family planning, then the average cost of treatment per fever may decline given the differences in costs of drugs and consumables.
- We do not include CHW compensation in these analysis – this will be the focus of a separate paper.

1. Source: <https://www.xe.com/currencyconverter/convert/?Amount=1&From=MGA&To=USD> [↑](#footnote-ref-1)
